# Jahn-Teller distortion and dissociation of CCl_4_^+^ by transient X-ray spectroscopy simultaneously at the carbon K- and chlorine L-edge†

**DOI:** 10.1039/d2sc02402k

**Published:** 2022-07-19

**Authors:** Andrew D. Ross, Diptarka Hait, Valeriu Scutelnic, Eric A. Haugen, Enrico Ridente, Mikias B. Balkew, Daniel M. Neumark, Martin Head-Gordon, Stephen R. Leone

**Affiliations:** Department of Chemistry, University of California Berkeley 94720 CA USA diptarka@stanford.edu; Chemical Sciences Division, Lawrence Berkeley National Laboratory Berkeley 94720 CA USA; School of Physics, Georgia Institute of Technology Atlanta 30332 GA USA; Department of Physics, University of California Berkeley 94720 CA USA

## Abstract

X-ray Transient Absorption Spectroscopy (XTAS) and theoretical calculations are used to study CCl_4_^+^ prepared by 800 nm strong-field ionization. XTAS simultaneously probes atoms at the carbon K-edge (280–300 eV) and chlorine L-edge (195–220 eV). Comparison of experiment to X-ray spectra computed by orbital-optimized density functional theory (OO-DFT) indicates that after ionization, CCl_4_^+^ undergoes symmetry breaking driven by Jahn–Teller distortion away from the initial tetrahedral structure (T_d_) in 6 ± 2 fs. The resultant symmetry-broken covalently bonded form subsequently separates to a noncovalently bound complex between CCl_3_^+^ and Cl over 90 ± 10 fs, which is again predicted by theory. Finally, after more than 800 fs, L-edge signals for atomic Cl are observed, indicating dissociation to free CCl_3_^+^ and Cl. The results for Jahn–Teller distortion to the symmetry-broken form of CCl_4_^+^ and formation of the Cl–CCl^+^_3_ complex characterize previously unobserved new species along the route to dissociation.

## Abstract 2

Dynamics of CCl_4_^+^ prepared by 800 nm strong-field ionization, as studied with X-ray transient absorption spectroscopy (XTAS) and quantum chemical calculations.

## Graphical abstract

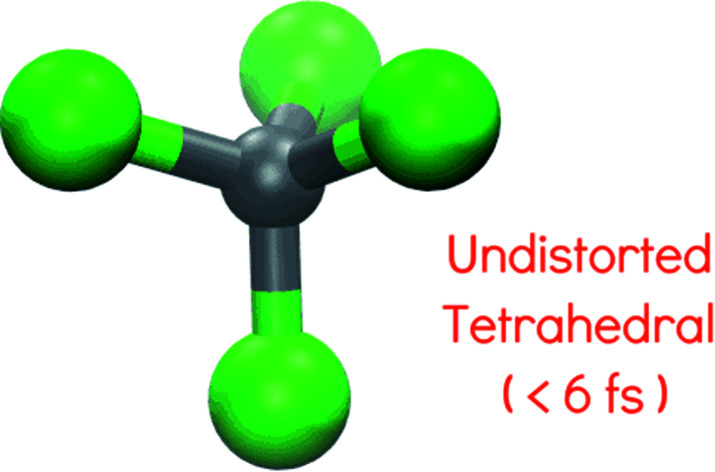

## 1 Introduction

The Jahn–Teller (JT) theorem^1^ states that degenerate electronic states in non-linear molecules cannot be minima of energy *vs.* nuclear positions and will therefore undergo nuclear displacements that break the degeneracy. This is often observed in highly symmetric molecules with partial occupation of symmetry equivalent orbitals. A prototypical example of JT distortion is CH_4_^+^, which arises from ionization from the triply degenerate highest occupied molecular orbital (HOMO) of CH_4_. The resulting cation rapidly distorts away from the tetrahedral (*T*_d_) geometry of the neutral to a *C*_2v_ symmetry structure^2,3^ with two long and two short CH bonds. Baker *et al.*^4^ have attempted to measure the time required for the JT process *via* comparison of high harmonic generation in CH_4_^+^ to CD_4_^+^, where they report a difference between the relaxation times of geometries of the two molecules up to 1.6 fs with sub-cycle resolution. However, information about longer times was limited by the wavelength of the laser. Recently, Gonçalves *et al.*^5^ calculated the JT timescale in CH_4_^+^ and found that distortion to *C*_2v_ occurs as early as 7 fs, while the heavier CD_4_^+^ takes longer to relax to the same structure.

Ionization of halogenated methanes is expected to lead to slower (and thus easier to measure) JT distortion, due to the larger substituent masses. However, JT distortion has not been observed for such systems, as the resulting cations are quite short-lived and therefore difficult to study.^6^ Indeed, it has been assumed that dissociation of the *T*_d_ conformation of CCl_4_^+^ to CCl_3_^+^ and Cl occurs without any intermediates,^7,8^ as no stable ion is produced by direct photoionization.^9^ Theoretical work^8,10^ has suggested a stable structure of the form [Cl_3_C–Cl]^+^, with one Cl 3.5 Å away from the C and weakly coordinating to it. However, there are no experiments that validate the existence of these possible CCl_4_^+^ complexes.

The instability of CCl_4_^+^ necessitates the use of a technique that is sensitive to changes in nuclear and electronic structure occurring within the first few femtoseconds after formation. X-ray Transient Absorption Spectroscopy (XTAS) is such a method, as its time resolution is only limited by the duration of the pump and probe laser pulses and femtosecond resolution is readily achievable.^14–16^ Following strong-field ionization by the pump, the X-ray pulse induces transitions from core levels to unoccupied orbitals, which ensures that XTAS is sensitive to the local environment of individual atoms in the probed molecular system.^15,17^ It is therefore well suited for probing possible JT distorted transient intermediates that may arise during a dissociation. Indeed, XTAS has previously been used to infer JT distortion in the benzene cation^18^ and ring opening in cyclohexadiene.^19^ Pertot *et al.*^20^ also employed XTAS at the C K-edge to study the dissociation of CF_4_^+^, reporting rapid dissociation to CF_3_^+^ and F within ∼40 fs.

While several aforementioned studies focused on the C K-edge, HHG X-ray pulses span a relatively broad energy range^14,21,22^ and can be used to study multiple atomic edges simultaneously. In particular, the Cl L_2,3_-edge (2p levels, ∼195–220 eV) is a natural complement to the C K-edge (1s level, ∼280–300 eV) for studying chlorinated methanes. Signal from two elements potentially allows for the identification of species whose spectra might be unresolvable or involve forbidden transitions at any one particular edge. It also permits observation of any Cl dissociation both from the perspective of the departing atomic Cl species and the remaining C-containing molecular fragment.

**Fig. 1:**
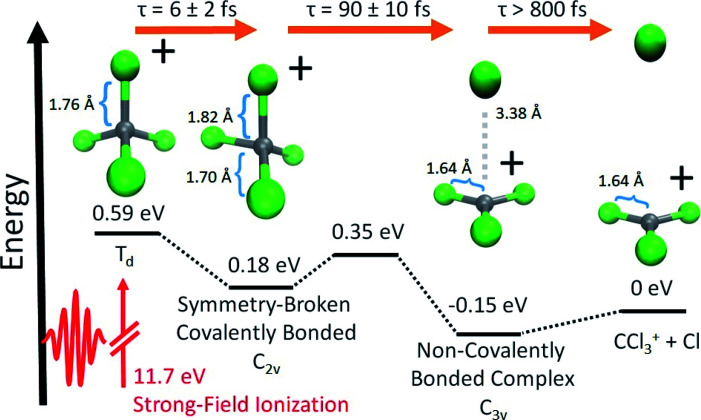
The sequence of computationally characterized intermediates for the dissociation of CCl_4_^+^ identified in this experiment. Geometries were optimized with ωB97M-V^11^/aug-pcseg-3.^12^ Zero-point energies were computed at the same level of theory. Electronic energies at these geometries were subsequently found using CCSD(T)^13^ extrapolated to the complete basis set (CBS) limit. The structures of the closest local minima are used for the symmetry-broken covalently bonded form and non-covalently bonded complex; although, the experiment samples a large range of nuclear configurations, due to vibrational energy. The energy is set to 0 for the dissociated ion. Time constants shown are gathered from the ΔOD data in Fig. 2b and c and their lineouts (as described in the ESI†). We refer to the symmetry-broken covalently bonded form as SBCB and non-covalently bonded complex as NBC, in subsequent figures and tables.

In this paper, we present the results of a time-resolved experimental and theoretical investigation of strong-field ionized CCl_4_, by tabletop XTAS simultaneously at both the C K-edge and the Cl L_2,3_-edge. We report evidence for a dissociation pathway of CCl_4_^+^, illustrated in Fig. 1, that is initiated by an ultrafast (∼6 fs) JT distortion driven symmetry breaking, away from the parent tetrahedral (*T*_d_) geometry to an ensemble of symmetry-broken covalently bonded structures where all four Cl atoms remain covalently bonded to C. All these bonded forms can be viewed as vibrationally excited *C*_2v_ CCl_4_^+^, as this geometry is predicted by theory to be the only stationary point on the cation potential energy surface with four C–Cl covalent bonds. Theory further indicates that the ions are only temporarily trapped in the bonded form, based on *ab initio* adiabatic quasiclassical trajectory (QCT) calculations and computed X-ray spectral features from orbital optimized density functional theory (OO-DFT^23^) that can be directly compared to experiment. The experiments corroborate the computed spectral features and identify the timescales for the initial symmetry breaking. Similarly, experiment and theory synergistically describe the evolution of the transient covalently bonded cation to a noncovalently bound complex between CCl_3_^+^ and Cl. The latter structure ultimately undergoes irreversible dissociation over relatively long timescales (∼800 fs). We also find evidence for a higher energy channel that results in CCl_3_ and atomic Cl^+^, which is expanded upon in the ESI.†

## 2. Methods

The tabletop experimental apparatus was previously described in ref. 14. We provide a brief summary that also touches on relevant upgrades. The pump pulse that induces strong-field ionization is centered at 800 nm, spectrally broadened, and compressed to about 6 fs in duration (as described in the ESI†). The maximum energy of the compressed pulse at the sample is 150 μJ at 1 kHz repetition rate. It is focused to 65 μm full width at half maximum (FWHM) to achieve an electric field with peak intensity up to 3 ± 1 × 10^14^ W cm^−2^, as estimated by numerical calculations and by observed relative ionization rates of Ar, as explained in the ESI.†

The X-ray probe is generated by high harmonic generation (HHG), using a 10 fs pulse, centered at 1300 nm, focused into a semi-infinite gas cell filled with 2.3 bar He. This tabletop setup provides X-ray photons with energies up to 370 eV. The X-ray monochromator has 0.2 eV spectral resolution at the C K-edge and the experimental data are measured as a change in absorbance, or ΔOD, which is separated into spectral components by multivariate fitting, described in detail in the ESI.† The temporal cross-correlation of the experiment is measured to be 8 ± 2 fs by the autoionization in Ar L_2,3_ lines^24^ (as discussed in the ESI†). Experiments are performed over different time ranges to cover a wider range of dynamics; the shortest have a step size of 1 fs and extend to 80 fs, and the longest have variable step sizes and extend to 10 ps. Additionally, experiments are run with varying pump power, from ∼1−3 × 10^14^ W cm^−2^, to assess how the power affects the temporal dynamics and final states. These scans are taken as closely in time as possible to make the comparisons between scans more consistent. Timescales are extracted by fitting lineouts in time to unimolecular kinetics (as described in the ESI†). Each fit includes convolution with a Gaussian of 8 ± 2 fs FWHM to account for the cross-correlation of the experiment, using the 95% confidence intervals as error bars. CCl_4_ was obtained from Sigma-Aldrich at 99.5% purity and was vaporized by exposing the liquid to vacuum at room temperature. The CCl_4_ was probed in a finite gas cell with a 4 mm pathlength with a foreline pressure of 12 mbar.

Quantum chemical calculations were performed with the Q-Chem 5 software.^25^ Structures were optimized with the ωB97M-V^11^ density functional and the aug-pcseg-3 (ref. 12) basis set. Zero-point energies were found at the same level of theory. Relative ground state electronic energies at the optimized geometries were computed with CCSD(T)^13^ extrapolated to the complete basis set (CBS) limit, as detailed in the ESI.†*Ab initio* adiabatic trajectory calculations on CCl_4_^+^ were performed with ωB97M-V/aug-pcseg-1, starting from the equilibrium CCl_4_*T*_d_ structure and with quasiclassical velocities^26^ for nuclei (this ensures each normal mode of the neutral species has the associated zero point energy). A total of 256 trajectories (out of the 512 possible ones for the CCl_4_ molecule with 9 normal modes) were run. The trajectory calculations did not incorporate an external electric field and thus can only provide a first estimate of timescales that can be compared to experiment.

X-ray absorption spectra were simulated with OO-DFT,^23^ utilizing the SCAN^27^ functional, aug-pcX-2 basis^28^ on the site of core-excitation, and aug-pcseg-2 basis^12^ on all other atoms. This approach has been shown to be accurate to ∼0.3 eV root-mean-squared error for the core-level spectra of electronic ground states of both closed-shell^29^ and open-shell^30^ species, without any need for empirical energy translation of the spectra. Ref. 29 and 30 provide detailed protocols for running OO-DFT calculations. Excited state orbital optimization was done with the square gradient minimization (SGM^31^) and initial maximum overlap method (IMOM^32^) algorithms, for restricted open-shell and unrestricted calculations, respectively.

## 3. Results and discussion

### 3.1 General features of experimental spectrum

**Fig. 2:**
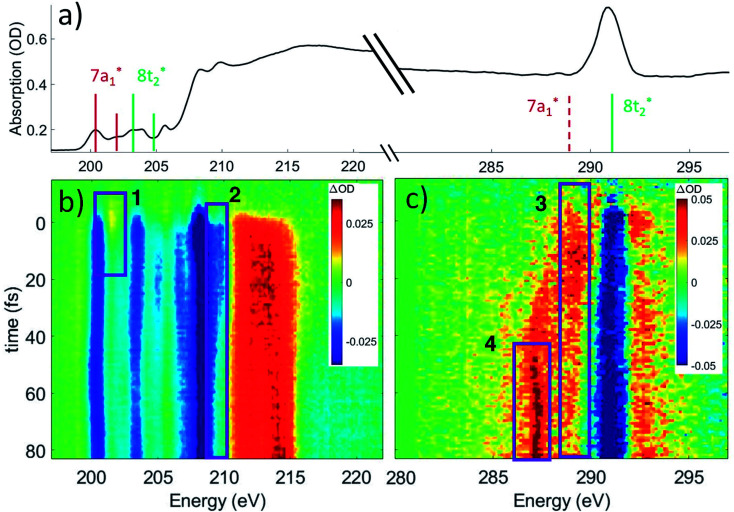
(a) Static absorption spectrum of neutral CCl_4_ at the Cl L_2,3_- and C K-edges, dominated mostly by transitions to the singly degenerate 
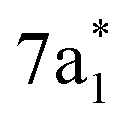
 and triply degenerate 
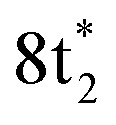
 levels. The 
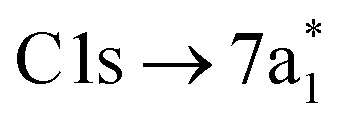
 transition is dipole forbidden and absent in the static spectrum. (b and c) ΔOD data from the highest pump power (∼3 × 10^14^ W cm^−2^) for the Cl L_2,3_-edge (b) and C K-edge (c). Positive time corresponds to 800 nm pump first, while negative time is X-ray probe first. Negative ΔOD represents depletion of neutral CCl_4_, while positive ΔOD features indicate presence of new species that absorb stronger than the parent in that region. Prominent transient features are labeled 1 (201.7 eV), 2 (209.5 eV), 3 (289.2 eV) and 4 (287.1 eV). A discussion of the features and their assignments is presented in the text. Additional data sets are shown in the ESI.†

The ground state static spectrum of CCl_4_ is presented in Fig. 2a. The Cl L_2,3_-edge comprises a number of peaks, several of which overlap, largely due to the C l2p spin–orbit splitting of 1.6 eV. The dominant features correspond to excitations to the 
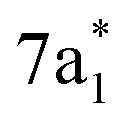
 and 
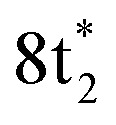
 symmetry adapted linear combinations (SALCs) of 
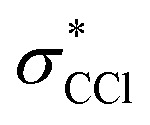
 antibonding orbitals. Transitions from the L_3_ (2p_3/2_) level to 
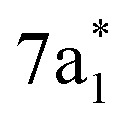
 and 
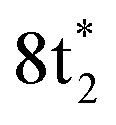
 occur at 200.4 eV and 203.2 eV respectively.^33^ The corresponding L_2_ (2p_1/2_) transitions occur at 202.0 eV and 204.8 eV. All of these excitations are dipole allowed. Transitions to Rydberg levels lead to additional peaks (such as the one at 205.5 eV (ref. 33)), and a rising ionization edge begins and persists around 207.5 eV. We note that the L_3_ ionization energy of CCl_4_ from X-ray photoelectron spectroscopy is 207 eV.^34^

In contrast, the C K-edge spectrum of neutral CCl_4_ consists of a single intense peak at 290.9 eV,^35^ followed by a rising edge at ∼295 eV. The 290.9 eV peak arises from the 
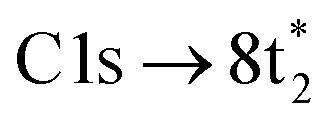
 excitation, as the transition to the lower energy 
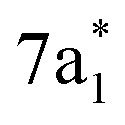
 orbital is dipole forbidden.^35^ OO-DFT predicts the 
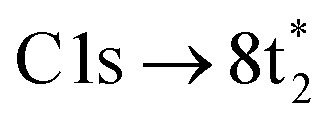
 excitation to be at 290.8 eV, in excellent agreement with experiment, and the forbidden 
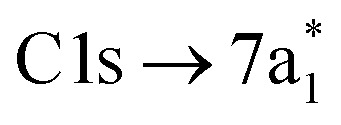
 transition is predicted at 289.0 eV. The HHG process leads to lower photon flux at the higher C K-edge energy range than the Cl L-edge, contributing to less signal-to-noise in the former. There is also significant absorption from the tail of the chlorine edge in the experimental spectrum, which further reduces the signal-to-noise at the C K-edge.

Strong-field ionization by the pump pulse induces dynamics that leads to differences in absorption (ΔOD), as shown in Fig. 2b and c. The many spectral overlaps at the Cl edge (Fig. 2b) make it difficult to identify individual signals in the ΔOD data. Negative signal from ground state bleach is predominant from 200 to 210 eV with positive signal appearing in the 211–216 eV range, possibly due to a positive local charge on the chlorines leading to a net blue shift. The C edge is easier to resolve, with only a ground state bleach at 290.8 eV, and positive absorption at both higher (∼292 eV) and lower (287–290 eV) energies.

Within these broad experimental signals, several notable features can be observed. The first is a small positive feature at the Cl edge, labeled feature 1 in Fig. 2b. This feature appears transiently at early times and rapidly decays within a few femtoseconds. The second is at 209.5 eV, feature 2, which initially drops to a negative ΔOD but decays back towards zero values on the timescale of 100 fs. A similar timescale is observed in the decay of feature 3, at 289.2 eV, which suggests that the two decays measure the same process. Additionally, the positive signal of feature 3 initially rises on the same time scale as the decay observed in feature 1, suggesting that the early time behavior of features 1 and 3 reflect the same process. A fraction of feature 3 undergoes a continuous evolution (289.2 to 287.1 eV) to feature 4 at 287.1 eV after ∼20 fs. Feature 4 continues to grow after that time, concurrent with the decay of feature 3. Finally, at delays of several hundred femtoseconds to a few picoseconds, much longer times than those shown in Fig. 2, a series of sharp spectral features between 204 and 207 eV become resolvable from the broader features, shown more clearly in Fig. 6 and described in Section III.†

Based on the three timescales involved in the evolution of the noted features, few-femtoseconds, tens of femtoseconds, and several hundred femtoseconds, we conclude that three separate processes are responsible for the dynamics of the dissociation of CCl_4_^+^. In order to better understand the nature and quantitative times of these processes, these features are averaged and fitted along the time axis, and assignments are made by comparison to theory, as discussed below. In particular, we note that our probe would not be able to directly track a hole in the Cl lone pair levels of pure 3p character. The Cl 2p → 3p transition is dipole forbidden, and the C 1s → Cl 3p process involves local sites on distinct atoms with essentially no overlap. Changes in electronic and nuclear structure arising from such Cl 3p holes would however lead to other transient features, as discussed later. Transitions from Cl 2s levels (L_1_ edge) to a 3p hole are dipole allowed. However Cl L_1_ edge transition peaks are very broad and not very intense (as shown through an example in the ESI†). We therefore did not pursue analysis in that regime (∼270 eV).

### 3.2 General considerations for C K-edge

Any distortion away from *T*_d_ geometries would alter the symmetry of the 
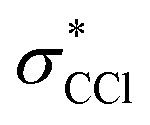
 levels, which would no longer form the 
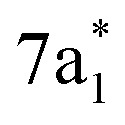
 and 
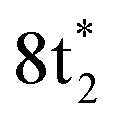
 SALCs. The most general symmetry-broken case would be of *C*_1_ symmetry, with four unequal bond lengths. This leads to four nondegenerate σ* levels that permit dipole allowed transitions from the C 1s level, although intensities will be lower for the MOs with greater C 2s character. The shorter bonds would feature stronger C–Cl interactions, leading to higher energy σ* MOs, while the longer bonds would conversely lead to lower energy σ* levels. In particular, the lowest energy 
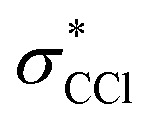
 level is expected to be dominated by the longest C–Cl bond, and therefore the lowest energy C K-edge feature in the transient absorption spectrum should correspond to this bond. Conversely, the shortest bonds should lead to the highest energy absorption feature. More symmetric configurations can permit multiple bonds to make comparable contributions to any given σ* MO, preventing assignment of a transition to one particular bond. In general, however, the longest bonds should lead to the lower energy features in the C K-edge spectrum, while the higher energy features should arise from shorter bonds. However, there is no such simple rule of thumb available for the Cl L-edges, due to the complexity of the spectrum.

### 3.3 Jahn–Teller distortion of CCl_4_^+^

**Fig. 3:**
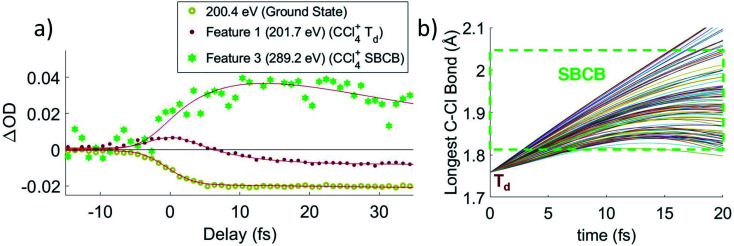
(a) Averaged lineouts of features 1 and 3 in the first 35 fs. Fitting including the 8 ± 2 fs cross-correlation gives a lifetime of 6 ± 2 fs and 7 ± 3 fs for the decay and rise of features 1 and 3, respectively. (b) The longest C–Cl bond length for a set of 100 trajectory calculations, because that bond will correspond to the lowest energy absorption at the C K-edge. It shows that the bond elongates into the symmetry-broken covalently bonded form (labeled SBCB) rapidly, assisting the assignment that feature 1 corresponds to the *T*_d_.

Lineouts of the few-femtosecond process are shown in Fig. 3a. These show the decay of feature 1 in 6 ± 2 fs and the rise of feature 3 in 7 ± 3 fs. These lifetimes, along with those extracted from the other fits, are compiled in Table 1 (with further details about the fits being provided in the ESI†). The ground state bleach at 200.4 eV from depletion of the neutral CCl_4_ represents the instrument response function (*i.e.*, the temporal broadening introduced by the experiment). Comparison of features 1 and 2 to the ground state bleach in Fig. 3a show these lifetimes are significant beyond the experimental cross-correlation.

**Table 1:** Time constants for each of the fits shown in the figures. Further details about the fits are provided in the ESI

Feature energy (eV)	Assigned transition	Time constant, *τ* (e^−1/*τ*×*t*^) (fs)
1 (201.7 eV)	*T*_d_ → SBCB	6 ± 2
3 (289.2 eV)	*T*_d_ → SBCB	7 ± 3
2 (209.5 eV)	SBCB → NBC	90 ± 10
3 (289.2 eV)	SBCB → NBC	80 ± 30
4 (287.1 eV)	NBC appearance	*τ*: 50 ± 20 delay: 23 ± 8
204.2, 206.6 eV	Atomic Cl appearance	800 ± 200
214.7 eV	Cl^+^ appearance	*τ*: 85 ± 10 delay: 37 ± 6

We assume the strong-field ionization process abruptly populates a CCl_4_^+^ cation state or states in a Franck–Condon-like manner, and those states then undergo geometry changes. The states may also have significant internal vibrational excitation. The lowest vertical ionization energy of CCl_4_ is 11.7 eV,^36^ corresponding to loss of an electron from the non-bonding *t*_1_ SALC of Cl 3p orbitals and forming the cation X state.^9,36,37^ The electron hole is thus essentially of pure Cl 3p character, and the C 1s → hole transition is consequently of negligible oscillator strength. Indeed, the computed C K-edge XAS of *T*_d_ CCl_4_^+^ is quite similar to neutral CCl_4_, due to the extensive delocalization of the hole over all four Cl atoms. None of the features of the C K-edge experimental spectrum can therefore be unambiguously assigned to *T*_d_ CCl_4_^+^. At the Cl L_2,3_-edges, the hole density should blue shift the absorption spectrum,^38^ and we make a tentative assignment of feature 1 to *T*_d_ CCl_4_^+^.

From theory, the closest local minimum to the *T*_d_ starting structure is a *C*_2v_ symmetry distorted tetrahedron with two long (1.82 Å) and two short (1.70 Å) C–Cl bonds. The calculations indicate that a *C*_2v_ form of CCl_4_^+^ is lower in energy by 0.4 eV, and there is no energy barrier between this minimum and the initial *T*_d_ geometry. This energy stabilization is smaller than the 1.5 eV stabilization observed for the analogous CH_4_ structure.^3^ This result is unsurprising as the CCl_4_ ionization is from Cl lone-pairs while the CH_4_ electron loss is from bonding orbitals. The energy stabilization from JT distortion will be available to the vibrational modes of the ion, so many different nuclear configurations will be accessible around the *C*_2v_ local minimum configuration. This range of configurations will be referred to as the symmetry-broken covalently bonded form of CCl_4_^+^ (or covalently bonded, for simplicity).

**Fig. 4:**
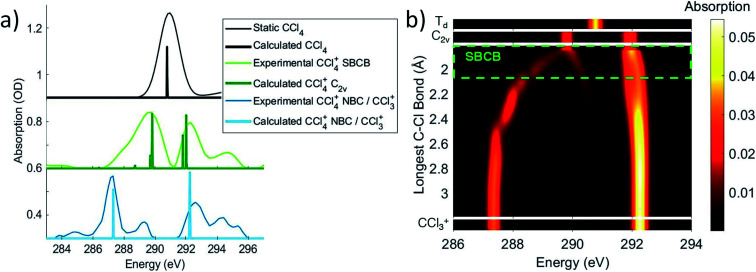
(a) The experimental features 3 and 4 as well as the neutral static are disambiguated and corrected for ground state bleach to obtain an absorption spectrum, using a method described in the ESI.† Notably, this shows that feature 3 ΔOD signal at 289.2 eV corresponds to an actual maximum at 289.8 eV. These spectra are compared to OO-DFT calculations for energies and oscillator strengths of the excitations (given by the peak position and heights, respectively). This allows for assignment of feature 3 to the symmetry-broken covalently bonded form (labled SBCB) and feature 4 to the noncovalent Cl–CCl_3_^+^ complex (labeled NBC) or CCl_3_^+^ moeity. (b) OO-DFT absorption spectra for various CCl_4_^+^ geometries. The third plot from the top shows absorption as a function of a single increasing bond distance (and other nuclear coordinates being optimized with this constraint). This shows splitting of absorption energies with the lower energies corresponding to the longest bond and higher energies to the shortest. The region of maximum bond extension without cleavage is roughly shown in the symmetry-broken covalently bonded region. These longer bonds account for the low energy tail in the covalently bonded experimental spectrum.

The OO-DFT C K-edge spectrum of the *C*_2v_ stationary point was computed in order to determine if the covalently bonded forms were contributing to feature 3. At the *C*_2v_ stationary point, all four σ* SALCs are nondegenerate and the C 1s transitions to these SALCs are all formally dipole allowed. The two lower energy SALCs correspond to the long C–Cl bonds, which is computed to lead to absorption at 289.8 eV, and the shorter bonds lead to higher energy σ* SALCs that are computed to absorb at ∼292 eV. A comparison of these energies in Fig. 4a with the absorption of feature 3, corrected for ground state bleach, shows that the energies match well. This validates the assignment that feature 3 arises from distorted CCl_4_^+^ with four covalent bonds.

In order to get an idea of the timescales of this distortion, Fig. 3b shows the longest C–Cl bond distance for a random subset of 100 calculated trajectories. The longest C–Cl bond is used here as a measure of distortion away from *T*_d_, for best comparison to the experimental X-ray absorption. It shows that at least one C–Cl bond rapidly elongates to the *C*_2v_ value of 1.82 Å at ∼5 fs on average.

The ∼5 fs time from the trajectories is comparable to the 6 ± 2 fs decay of feature 1 and the 7 ± 3 fs rise of feature 3. Both time constants should measure the same process, the lifetime of the CCl_4_^+^*T*_d_ geometry, with feature 1 more directly measuring the decay of the initial *T*_d_ state and feature 3 measuring formation of the distorted state. They confirm the rapidity of the JT process and the barrierless energy surface between the two. We also note that on changing the pump intensity, the lifetimes exhibited no power dependence beyond what is necessary for ionization within the error bounds.

### 3.4 Covalent bond breakage of CCl_4_^+^

The evolution of some fraction of feature 3 to the much lower energy feature 4 in the experimental spectrum suggests further C–Cl bond stretching. OO-DFT calculations confirm this, with Fig. 4b showing the absorption of energy in CCl_4_^+^ as a function of the longest C–Cl bond distance (with all other nuclear coordinates being optimized). Fig. 4b further explains the lower energy tail of the experimental covalently bonded form spectrum in Fig. 4a, as the experiment will sample many molecules spanning a wide range of covalently bonded geometries.

Feature 4 at 287.1 eV absorbs at the same energies as an extremely elongated C–Cl distance (∼3 Å) in Fig. 4a and b, suggesting that feature 4 corresponds to a CCl_3_^+^ moiety. Indeed, this feature corresponds to a transition to the unoccupied 2p orbital in CCl_3_^+^. This CCl_3_^+^ orbital originates from the lowest energy 
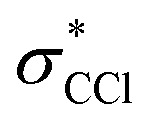
 level of the covalently bonded form, which has decreasing contribution from the dissociating Cl atom as the C–Cl distance increases. The observed spectrum is thus consistent with the theoretical prediction that the lowest energy forms of CCl_4_^+^ are noncovalently bound complexes between atomic Cl and CCl_3_^+^. The global energy minimum of CCl_4_^+^ is a complex with approximately *C*_3v_ symmetry, a nearly planar CCl_3_^+^ moiety having a Cl atom vertically above the C, at a distance of 3.4 Å. A *C*_s_ symmetry minimum with the atomic Cl coordinating to a bonded Cl in the CCl_3_^+^ moiety is also found at 0.02 eV above the minimum energy complex, with a Cl–Cl distance of 3.24 Å. Given the only slight energy preference for the minimum energy position of the Cl in the complex and the available vibrational energy, it is likely that the Cl does not stay at this position under experimental conditions and instead samples a wide range of locations around CCl_3_^+^. However, the experiment is not directly sensitive to the position of the noncovalently bound Cl. The large distances between CCl_3_^+^ and Cl for all such species in fact indicate that C K-edge transitions to valence orbitals would be essentially unaffected by the particulars of the noncovalent interaction.

An energy decomposition analysis (EDA^41^) calculation reveals that the −0.15 eV interaction energy between atomic Cl and CCl_3_^+^ in the minimum energy complex geometry is mostly (56%) from the polarization of the atom by the cation, with a much smaller amount (19%) arising from charge-transfer (the remaining 25% being permanent electrostatics, Pauli repulsion and dispersion). The system effectively acts as a charge-induced dipole complex. It is also worth noting that these noncovalent complex structures do not have any covalent Cl–Cl interactions, contrary to some early assignments.^42–44^

**Fig. 5:**
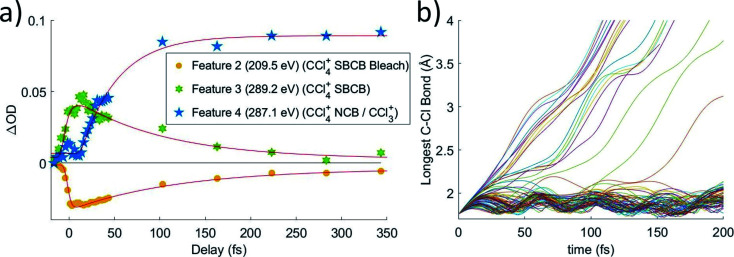
(a) Averaged lineouts of features 2, 3, and 4 out to 350 fs. Fitting gives decay times of features 2 and 3 in 90 ± 10 fs and 80 ± 30 fs, respectively. Feature 4 appears only after a delay of 23 ± 8 fs, followed by a growth signal with a lifetime of 50 ± 20 fs. Acronyms: SBCB = symmetry-broken covalently bonded CCl_4_^+^, NBC = non-covalently bonded Cl–CCl_3_^+^ complex. (b) A set of 100 trajectory calculations, showing the longest C–Cl bond distance. It shows a population that temporarily remains in the covalently bonded form with longest bonds going between 1.8 and 2.05 Å. It also shows a portion of ions have sufficient velocity in the appropriate chlorine to break the C–Cl covalent bond immediately, while others require additional time to redistribute the energy such that the bond may be broken. The trajectories show more population remaining in the covalently bonded form for a longer time, but the difference is likely due to excess vibrational energy in the experiment or another particular of the strong-field ionization process, which are not included in the trajectories. See Section IX of ESI† for a discussion about the long time behavior of trajectories.

The timescale of the symmetry-broken covalently bonded CCl_4_^+^ species to Cl–CCl_3_^+^ complex transition is shown through lineouts of features 2, 3, and 4 in Fig. 5a. This shows decay times of features 2 and 3 in 90 ± 10 fs and 80 ± 30 fs, respectively. Feature 4 appears only after a delay of 23 ± 8 fs, which implies that the nuclear motions take at least 23 fs to get to the point that the ion starts to resemble its dissociated form. After this delay, it has an additional lifetime of 50 ± 20 fs towards reaching its asymptotic value. Because of the delay from nuclear movement, the experimental time for the covalently bonded to noncovalent complex process is taken from the more precise feature 2, 90 ± 10 fs, and no pump power dependence is observed. This time is expected to be significantly longer than the JT distortion due to the computed barrier of 0.17 eV for this process. In varying the pump power, we do not observe a difference in the covalently bonded population decay times, possibly due to the large intensity already required to ionize the CCl_4_.

The trajectory calculations in Fig. 5b show that a portion of the ions continue their initial distortion and dissociate immediately, which is observed experimentally by the direct evolution of the feature 3 → feature 4 signal. Indeed, this regime of the experimental signal reports direct dynamics from the covalently bonded form to the complex for a fraction of the molecules. Another portion remains trapped in the covalently bonded form for longer than a vibrational period, showing longest C–Cl distances between 1.8 and 2.05 Å, which serves to define the symmetry-broken covalently bonded region. We however note that the trajectory calculations show a significant population persisting in the covalently bonded form for a longer time than the experimentally measured lifetime. This likely arises from the trajectory calculations not including the excess vibrational energy added by the pump pulse. The calculations only include the zero-point energy of neutral CCl_4_, leading to the covalently bonded form persisting longer than what is experimentally observed. A further discussion about the long time behavior of the trajectories is given in Section IX of the ESI.†

### 3.5 Atomic Cl dissociation

**Fig. 6:**
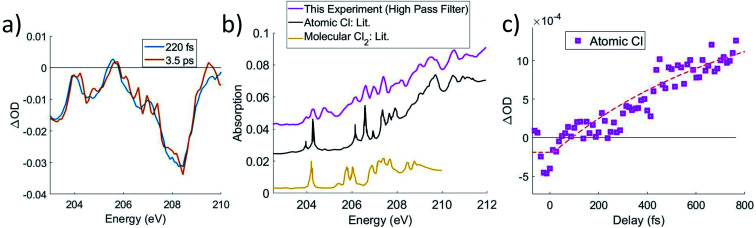
(a) Lineouts in energy for 220 fs and 2.5 ps at the Cl L_2,3_-edges. They show that although the overall shape and intensity of the ΔOD signal remains constant, sharp spectral features appear at longer delays. The difference between 3.5 ps and 220 fs is taken and added to an error function to obtain the spectrum in b. (b) A comparison of the sharp spectral features is made to atomic Cl^39^ and to molecular Cl_2_,^40^ which shows that the features match well with atomic Cl. (c) A high-pass spectral filter is applied to the ΔOD data and an average of the two most prominent lines at 204.2 and 206.6 eV is shown as a lineout against time. A fit is taken starting from *t* = 0, due to noise preventing a better determination of a start point. This fit with ∼3 × 10^14^ W cm^−2^ pump intensity has a lifetime of 800 ± 200 fs.

Although the C K-edge cannot distinguish between noncovalent complex and free Cl, the Cl L_2,3_ edge can make this distinction, as atomically sharp Rydberg lines corresponding to atomic Cl become evident upon completion of the dissociation, which is shown in Fig. 6a and b. Very little change is observed in the ΔOD spectra between 250 fs and 3.5 ps at either the C K-edge or chlorine L_2,3_-edge, other than the appearance of these sharp lines, which is consistent with the previous assignment of the noncovalent complex. While weakly bound in the complex, the atomic lines are broadened by a combination of two factors. Firstly, the transitions are already of low intensity due to their Rydberg character, the lowest energy lines being of 2p → 4s character. Secondly, the diffuse nature of the Rydberg levels means that the excitation energy would be sensitive to the precise position of the CCl_3_^+^ entity, and the flatness of the complex ground state potential energy surface permits a large range of accessible nuclear configurations. However, over time, the complex irreversibly dissociates, leading to atomic Cl lines at 204.2 eV and upward from 206 eV, which agrees with previous experimental absorption data.^39^ Thus, we can use the Cl atomic lines to track the time it takes for the complex species to completely dissociate to CCl_3_^+^ and Cl. We note that molecular Cl_2_ has absorption in this energy range as well;^40^ however, comparison to experimental atomic absorbance in Fig. 6b shows much better agreement with atomic Cl.

A high-pass filter is applied to the transient spectrum to separate the sharp atomic Cl lines from broader molecular signals. A simple exponential fit of these lines in Fig. 6c gives a lifetime longer than 800 fs. The exponential starts at time zero, despite the Cl signal not clearly departing from 0 until ∼300 fs. This may correspond to the minimum time required for atomic Cl to move far enough to show the Rydberg lines, although the signal-to-noise of this experiment is not sufficient to make that distinction.

The 800 fs time scale is much longer than the 90 fs observed for noncovalent complex formation, despite the excess of energy available to the system. However, the interactions and thus rate of energy transfer between the CCl_3_^+^ moeity and the neutral Cl are much weaker than a covalent bond. This situation is similar to van der Waals complexes, which sometimes show lifetimes exceeding milliseconds with vibrational energy in the molecular moeity.^45,46^ The 800 fs value is the fastest Cl formation time and was measured from the most intense pump pulse of 3 × 10^14^ W cm^−2^. Datasets collected at lower pump power show slower dissociation times, and some do not exhibit significant atomic Cl formation up to 3 ps. The noncovalent complex signal from feature 4 at 287.1 eV is still present, suggesting that the strong-field ionization may be capable of forming long-lived noncovalently bound CCl_4_^+^ complexes; although times longer than a few ps are outside the scope of this paper. It has been observed that otherwise unstable parent ions can be generated by few-cycle strong field ionizing laser pulses, such as tetramethyl silane and CS_2_,^47,48^ and an analogous complex may be a potential explanation of these signals. A quantitative comparison of the power dependence on this timescale was not carried out. We also note that the lifetime of a molecular species against collision with an electron (released by strong-field ionization) is on the order of ∼5 ps with our setup (as discussed in the ESI†), which is an additional factor to consider, but only at very long times.

## 4 The high intensity case and the formation of Cl^+^

While the observed dissociation to CCl_3_^+^ and Cl can be well explained by the intermediate forms and pathways discussed above, another channel appears to be present in the data. This channel is clearest at the chlorine L_2,3_-edge where very sharp lines with similar widths to the atomic Cl lines are observed at 214 eV with less obvious components at 212.6 eV, shown in the ESI.† These energies are in the range that the calculations predict for the Rydberg states (2p → 3d, 4s) of Cl^+^. These atomically sharp peaks begin to appear with a delay of 37 ± 6 fs relative to the onset of the main cationic CCl_4_ signal, and from that point, they show a time to grow in of 85 ± 10 fs for the best signal-to-noise dataset.

Low energy satellite features in the C 1s spectrum ∼285.5 eV are also observed, which can correspond to transitions to the singly occupied level of the CCl_3_ radical, computed to be at 285.5 eV. These simultaneous features appear to suggest the existence of a channel that results in CCl_3_ and Cl^+^. The formation of Cl^+^ is a much higher energy channel, with final energies about 4.8 eV higher than the normal dissociation channel. Cl^+^ formation has been observed in previous CCl_4_ ionization experiments in low quantities from electron impact,^49^ single-photon ionization^9,50^ and in higher quantities from strong-field ionization.^51^ Further discussion about Cl^+^ formation is provided in the ESI.†

## 5 Conclusions

In this paper, we have shown experimental and theoretical evidence for both a transient Jahn–Teller distorted symmetry-broken covalently bonded CCl_4_^+^, similar to the stable ion of CH_4_^+^, and a noncovalently bound complex between CCl_3_^+^ and Cl. Neither of these intermediates have been observed previously in experiments. A summary of each of the time constants experimentally extracted is shown in Table 1. The transition from the initial tetrahedral ion to the *C*_2v_ state occurs in 6 ± 2 fs, showing that the Jahn–Teller distortion is very fast, on the order of the duration of the pump laser pulse. It provides an order of magnitude estimate for other symmetric molecules undergoing JT distortion, which can aid chemical simulations as well as help predict the vibrational energies and nuclear dynamics of those other systems, especially given that this symmetry-broken covalently bonded intermediate was not assumed to exist prior to this work.^7,8^

The noncovalent Cl–CCl_3_^+^ complex is bound largely by the polarization of the neutral species, forming an ion-induced dipole complex with a lifetime much longer than the covalent bond cleavage time. The final dissociation time for free Cl or Cl^+^ seems to be slightly dependent on the strong-field power, but for the highest power used in this experiment, the lifetime is more than 800 fs (*vs.* 90 fs for noncovalent complex formation). This observed long lifetime is a potential explanation for otherwise unstable parent ions appearing in mass-spectra of ultra-short strong-field ionization.

## Data availability

Data are available upon request to the authors.

## Author contributions

A. D. R. and D. H. contributed equally to this work. A. D. R., V. S., E. A. H., E. R., and M. B. B. performed experiments. A. D. R. analyzed experimental data. D. H. performed calculations. S. R. L., M. H. G., and D. M. N. supervised the project. A. D. R. and D. H. wrote the manuscript, with inputs from all the authors. All authors reviewed the manuscript.

## Conflicts of interest

The authors declare the following competing interest: M. H.-G. is a part-owner of Q-Chem, which is the software platform in which the quantum chemical calculations were carried out.

## Supplementary Material

SC-013-D2SC02402K-s001

SC-013-D2SC02402K-s002

SC-013-D2SC02402K-s003

SC-013-D2SC02402K-s004

SC-013-D2SC02402K-s005

SC-013-D2SC02402K-s006

SC-013-D2SC02402K-s007

SC-013-D2SC02402K-s008

SC-013-D2SC02402K-s009
