# Correction: Jahn-Teller distortion and dissociation of CCl_4_^+^ by transient X-ray spectroscopy simultaneously at the carbon K- and chlorine L-edge

**DOI:** 10.1039/d2sc90172b

**Published:** 2022-08-24

**Authors:** Andrew D. Ross, Diptarka Hait, Valeriu Scutelnic, Eric A. Haugen, Enrico Ridente, Mikias B. Balkew, Daniel M. Neumark, Martin Head-Gordon, Stephen R. Leone

**Affiliations:** Department of Chemistry, University of California Berkeley 94720 CA USA srl@berkeley.edu; Chemical Sciences Division, Lawrence Berkeley National Laboratory Berkeley 94720 CA USA; School of Physics, Georgia Institute of Technology Atlanta 30332 GA USA; Department of Physics, University of California Berkeley 94720 CA USA

## Abstract

Correction for ‘Jahn-Teller distortion and dissociation of CCl_4_^+^ by transient X-ray spectroscopy simultaneously at the carbon K- and chlorine L-edge’ by Andrew D. Ross *et al.*, *Chem. Sci.*, 2022, https://doi.org/10.1039/d2sc02402k.

The authors regret that the corresponding author email address listed was incorrect. The correct email address is srl@berkeley.edu as shown in this correction article.

The Royal Society of Chemistry apologises for these errors and any consequent inconvenience to authors and readers.

## Supplementary Material

