# Supplementary material for: Jahn-Teller distortion and dissociation of CCl4+ by transient X-ray spectroscopy simultaneously at the carbon K- and chlorine L-edge
Source: Chem Sci. 2022 Jul 19;13(32):9310–20. doi: 10.1039/d2sc02402k (PMC9384822; doi:10.1039/d2sc02402k)
Supplement: SC-013-D2SC02402K-s002 [file SC-013-D2SC02402K-s002.pdf]

Electronic Supplementary Material (ESI) for Chemical Science.
This journal is © The Royal Society of Chemistry 2022

Electronic Supporting Information For Jahn-Teller Distortion and Dissociation of CCl_4^+ by Transient X-ray Spectroscopy Simultaneously at the Chlorine $L_{2,3}$- and Carbon K-Edges

Andrew D. Ross,[1,2] Diptarka Hait,[1,2] Valeriu Scutelnic,[1] Eric A. Haugen,[1,2] Enrico Ridente,[1] Mikias B. Balkew,[3,1] Daniel M. Neumark,[1,2] Martin Head-Gordon,[1,2] and Stephen R. Leone[1,2,4]

[1)] *Department of Chemistry, University of California, Berkeley, CA, 94720, USA*

[2)] *Chemical Sciences Division, Lawrence Berkeley National Laboratory, Berkeley, CA, 94720, USA*

[3)] *School of Physics, Georgia Institute of Technology, Atlanta, GA, 30332, USA*

[4)] *Department of Physics, University of California, Berkeley, CA, 94720, USA*

(Dated: 6 June 2022)

CONTENTS

I. Treatment of Experimental Data 3

II. Method of Fitting Lineouts to Unimolecular Kinetics 3

III. Pump Pulse and Cross-Correlation 6

IV. On the Presence of CCl_4^+ Excited States 7

V. Electric Field Effects Near Time Zero 11

VI. Additional Channel Leading to Cl^+ Formation 11

VII. Time Constant Changes with Varying Pump Intensity 14

VIII. Calculated Geometries and Energies of Intermediates 16

IX. Calculated Normal Modes 17

X. Calculated C K-edge Transitions and Assignments 18

XI. Comment on Quasiclassical Trajectory Calculations 21

XII. Frequency of molecule-molecule and molecule-ion collisions 22

XIII. Separation of Spectral Components from Experimental Data by Multivariate Fitting 23

XIV. Method of Intensity Estimation by Ar Ionization 27

XV. Further Analysis of Distortion from Trajectory Calculations 28

XVI. Additional CCl_4^+ Data 30

References 36

I. TREATMENT OF EXPERIMENTAL DATA

The experimental data was calibrated from CCD pixel to energy by comparison to known energies in SF_6[1], Ar[2], and CO_2[3]. Transient data was collected in pump-on/pump-off configuration to collect a change in absorption, ΔOD:

$$\Delta\mathrm{OD} = \left(-\log_{10}\left(\frac{I_{on}}{I_0}\right)\right) - \left(-\log_{10}\left(\frac{I_{off}}{I_0}\right)\right) = -\log_{10}\left(\frac{I_{on}}{I_{off}}\right) \quad (1)$$

where I_{on} is the spectrum measured with the pump on and I_{off} is the spectrum with pump off. The reference or unabsorbed spectrum, I_0, is eliminated through use of the logarithms and is therefore not collected at each iteration. The data was filtered using edge reference filtering[4]. Separate edge referencing was done for the chlorine $L_{2,3}$-edges and the carbon K-edge.

II. METHOD OF FITTING LINEOUTS TO UNIMOLECULAR KINETICS

The key transformation being studied in this work (as depicted in Fig. 1 of the main manuscript) is the following three step process

$$\mathrm{T}_d \xrightarrow{\mathrm{k_1}} \mathrm{SBCB} \xrightarrow{\mathrm{k_2}} \mathrm{NBC} \xrightarrow{\mathrm{k_3}} \mathrm{CCl_3}^+ + \mathrm{Cl} \quad (2)$$

where the symmetry-broken covalently bonded form is abbreviated as SBCB, and the non-covalently bonded $Cl{-}{-}{-}CCl_3^+$ complex is abbreviated as NBC. These processes are assumed to be unimolecular (due to low gas pressure, as discussed later in Sec XII) and irreversible. They have associated lifetimes $\tau_1 = \frac{1}{k_1}, \tau_2 = \frac{1}{k_2}$, and $\tau_3 = \frac{1}{k_3}$, which were reported to be 6±2 fs, 90±10 fs and > 800 fs in the main manuscript. Herein, we discuss how these values were obtained via fitting to lineouts.

It superficially seems natural to directly fit to the sequential, three-step reaction. However, we instead separate these processes into two stages

$$\mathrm{T}_d \xrightarrow{\mathrm{k_1}} \mathrm{SBCB} \xrightarrow{\mathrm{k_2}} \mathrm{NBC} \quad (3)$$

and

$$\mathrm{NBC} \xrightarrow{\mathrm{k_3}} \mathrm{CCl_3}^+ + \mathrm{Cl} \quad (4)$$

They are assumed to be effectively decoupled from each other. The rationale for this separation is described at the end of this section.

The first stage can be modeled by rate equations:

$$\frac{d[\mathrm{T}_d]}{dt} = -k_1[\mathrm{T}_d] \tag{5}$$

$$\frac{d[\mathrm{SBCB}]}{dt} = k_1[\mathrm{T}_d] - k_2[\mathrm{SBCB}] \tag{6}$$

$$\frac{d[\mathrm{NBC}]}{dt} = k_2[\mathrm{SBCB}] \tag{7}$$

in terms of populations $[\mathrm{T}_d]$, [SBCB], and [NBC]. Integrating the above differential equations[5], we find that the populations are given by:

$$[\mathrm{T}_d] = [\mathrm{T}_d]_0 \times e^{-k_1 t} \tag{8}$$

$$[\mathrm{SBCB}] = \frac{k_1[\mathrm{T}_d]_0}{k_2 - k_1} \times (e^{-k_1 t} - e^{-k_2 t}) \tag{9}$$

$$[\mathrm{NBC}] = [\mathrm{T}_d]_0 - [\mathrm{T}_d] - [\mathrm{SBCB}] \tag{10}$$

For fitting the lineouts, we fit the absorbances of the T_d, SBCB, and NBC at the energy of the lineout, as well as the rates k_1 and k_2, represented as the lifetimes $\tau_1 = \frac{1}{k_1}$ and $\tau_2 = \frac{1}{k_2}$.

This model makes the following assumptions:

1. The transition between each of the ion forms are unimolecular and irreversible, which is likely, given that only a single molecule is involved and all steps are energetically favorable.

2. No other species or processes are involved in significant amounts (as discussed later).

3. Fitting based on the differential equations assumes that individual molecules transition between two configurations at a rate that is much faster than the rate of the reaction. In other words, the lifetime of any intermediate form (along the reaction path) is much smaller than the reported lifetimes. This is rather unlikely to be the case for the processes in the first few tens of femtoseconds. However, the model can still be used as a measure of how similar the average population of the molecules are to each of the two limiting configurations (i.e. 'reactant' and 'product') if the change in absorption for any intermediate form can be found via linear interpolation between the limiting configurations, at a given energy lineout. A case where this assumption would not

apply is the absorption of the NBC at 287 eV. Zero absorption is observed therein until ~23 fs, due to the fact that no absorption can occur until a C-Cl bond distance of ~2.5 Å is reached. For this reason, lineouts are chosen at energies where absorption is present for all species of interest.

The fitting also assumes that $\tau_1 < \tau_2$ even though the shape of the intermediate population [SBCB] would be similar in the opposite case. This assumption is made, based on the theoretical prediction that the Jahn-Teller distortion to SBCB should be a barrierless process, where the bond cleavage to NBC should have a barrier of 0.2 eV. Additionally, the data shows the shift of absorbance from 289.6 eV to 287.1 eV starting around 14 fs, which would only be expected if the Jahn-Teller distortion to SBCB were completed relatively quickly.

Our fits generally agree well with the data, which shows that the exponential nature of the fits is sufficient, within the signal-to-noise of the experiment.

The NBC $\xrightarrow{\mathrm{k_3}}$ CCl_3^++Cl process is more difficult to fit, as the NBC and CCl_3^+ have rather similar absorption profiles, especially at the energies chosen for fitting k_1 and k_2. The signals associated with the NBC dissociation to form free Cl are very weak when integrated across many energies. However, fitting can be done via applying a high pass filter, which eliminates the broad molecular peaks while leaving behind atomic Cl signals. The growth in these signals (at 204.2 eV and 206.6 eV) is fit to:

$$[\mathrm{Cl}] = [\mathrm{Cl}]_\infty \times (1 - e^{-k_3 t}) \tag{11}$$

The decoupling of this last step from preceding steps can be justified by noting that the resulting $\tau_3 = \dfrac{1}{k_3} \sim 800$ fs is considerably longer than the previously obtained τ_1, τ_2. Therefore, Eqns 8 and 9 indicate $< 10^{-4}$ fraction of the molecules are left in either the T_d or SBCB configurations. Thus, NBC formation is effectively complete on the timescale of Cl dissociation. Additionally, the energies of the lineouts for the k_1 and k_2 kinetics were chosen such that the change in absorption to the last step is zero, and the high-pass filter for the atomic Cl eliminates the effects of the first two processes. These processes can therefore be neglected while modeling the last step.

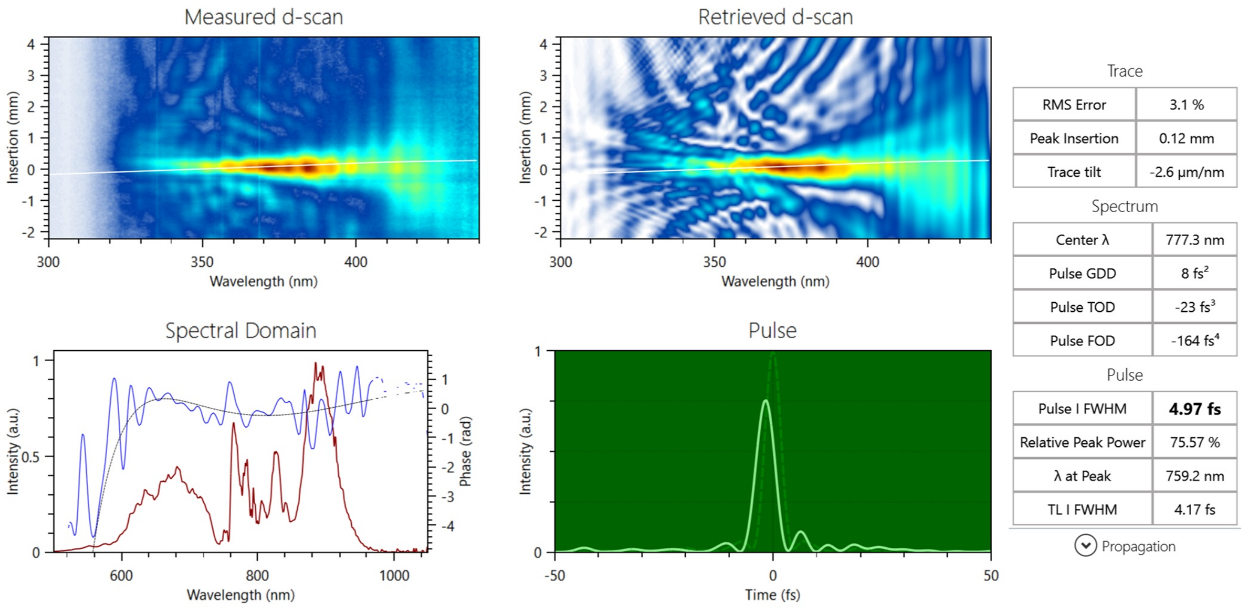

FIG. 1. Output of the d-scan software[6,7]. Top-left: Measured, calibrated SHG d-scan as a function of fused-silica insertion. Top-right: Numerical retrieval of measured d-scan. Bottom-right: Measured input spectrum (red) and retrieved spectral phase (blue). 4th order polynomial fit of the phase (black). Bottom-right: Retrieved temporal intensity profile of the measured pulse (light-green) and temporal intensity of the transform limited pulse (dashed green).

III. PUMP PULSE AND CROSS-CORRELATION

The pump pulse was characterized by a Sphere Ultrafast Photonics d-scan, which retrieves the short pulse duration and the temporal structure of the pulse[6,7]. The measurements were taken outside of the vacuum chambers with compensation glass and air to mimic the path into the vacuum chambers. Over the many days of measurements, the shortest duration was found to be 3.75 fs and the longest was 6 fs FWHM. The latter is reported in the main text, to be conservative. A representative retrieval is shown in Fig. 1, which shows a 5 fs pulse. No long temporal tails or significant pre or post pulse effects are observed. The pump pulse is linearly polarized.

The cross-correlation of the experiment was determined by suppression of atomic absorption lines in Ar by perturbation of autoionization[2,9]. The measurement was taken as transient absorption with 1 fs steps, $\sim 1 \times 10^{13}$ W/cm^2 pump intensity, and 1000 ms camera integration. The data was filtered by the method described by Ott et.al. to increase the

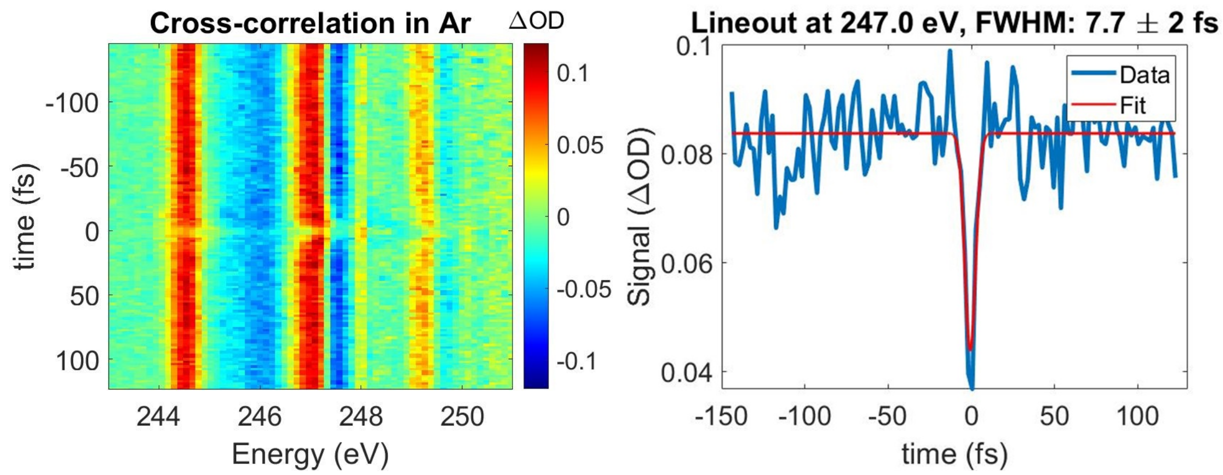

FIG. 2. Cross-correlation as determined by suppression of Ar $L_{2,3}$ lines. Left: Transient ΔOD plot, using a high-pass filter[8], the positive (red) are the sharp features of the unperturbed absorption. The feature at time zero corresponds to interaction with the pump pulse. Right: A lineout is taken at the $2p_{3/2}^{-1}3d$ line (247.0 eV) and fit to a Gaussian, resulting in a fit of 8 ± 2 fs cross-correlation.

signal-to-noise ratio[8]. The suppression of the $2p_{3/2}^{-1}3d$ line was fitted to a Gaussian and the FWHM was found to be 8 ± 2 fs, as shown in Fig. 2. This cross-correlation covers many broadening factors, such as duration of pump and probe pulses, timing jitter between pump and probe pulses, and unlocked carrier-envelope phase. These factors will lead to a Gaussian broadening of the signal in the time domain and are included in all kinetic fits in the paper.

IV. ON THE PRESENCE OF CCl_4^+ EXCITED STATES

In the main text, comparisons to the simulated spectra are done for the ground (X) state of CCl_4^+. A potential concern is whether excited states of CCl_4^+ could provide an alternative explanation or whether such states are present at all. During the strong-field ionization, the large amount of available energy could potentially lead to excited states of the cation. A simple picture of strong-field ionization assumes that it leads to ionization into the ground state of the cation[10,11]; however, excited states are possible through mechanisms such as recollision of the electron due to the electric field[12,13]. Other studies have shown that ionization from lower electronic levels can be enhanced, based on the orientation of the molecule, relative to the polarization of the electric field[14,15]. Additionally, there are other pump schema used in other literature that can reduce or disambiguate the effects of such

excited states. These pump schema can include: scanning the angle of the polarization or changing the ellipticity of the polarization[16], modification of the pump pulse duration or spectral phase[17,18], changes in wavelength of the pump pulse[19], or use of pre-pulses to orient the molecule or prepare it in a vibration or electronic state[20,21]. This paper only uses a linearly polarized pump laser and measures the differences in intensity, so the presence or lack thereof of excited states must be infered from ΔOD data. It is difficult to completely rule out such states, but they likely do not contribute significantly to the $CCl_3{}^+$ + Cl channel. They do however likely lead to Cl^+ formation as a side channel. It is also possible that the molecule is ionized to such higher energy excited states and quickly relaxes to the ground state[22]. However, our method is not able to distinguish these possibilities, and it would not strongly affect the mechanisms described.

We consider four classes of excited states, and analyze their potential relevance via insight from quantum chemical calculations:

1. **Ionization from a deeper level:** The A and B states of the cation are relatively readily accessible[23], being ionizations from other 3p lone pair SALCS (t_2 and e) at 12.5 and 13.5 eV respectively. For each of these, there would be no perceptible difference in the Franck-Condon absorption at the C K-edge, because the electron is coming from purely Cl orbitals. Optimization of the A state starting from the initial T_d geometry leads to no local minima, but goes straight to the NBC form (unlike the X state, which leads to the SBCB form). We observe a delay in the formation of the 287.1 eV NBC feature, which would indicate that the bond is not dissociating immediately, but going through the SBCB forms.

   The B state optimizes to a D_{2d} minima (all four bonds of equal length, but angles deviating from T_d) and indeed experimental vibrational spectrum for this form has been observed in photoionization experiments[23], indicating it can exist in a metastable form. However, this D_{2d} geometry B state is computed to absorb very close to the static spectrum of neutral CCl_4 at the C K-edge and is thus invisible to that probe.

   The C (∼16 eV) and D (∼20 eV) states are ionizations from the t_2 and a_1 bonding SALCs (σ_{CCl} bonding orbitals), respectively, and thus would have C K-edge excitations at ∼278-282 eV. Only the C state should be bright, as the transition to the D state hole is dipole forbidden at the C K-edge. Both the C and D states would also strongly

absorb in the Cl L-edge at 187-190 eV, which is consistently zero in the ΔOD data. The absence of any features there suggest lack of holes in the bonding levels in general.

Beyond the C and D states, ionization from the Cl 3s lone pairs is a possibility, albeit at rather high (∼ 27 eV) energies. This should lead to bright absorption at low Cl L edge energies ∼ 180 eV that do not appear in the experiment, although such states would be dark to the C K-edge probe. Subsequent ionizations would occur from the L-edge itself (∼ 207 eV) and are unfeasible with the pump used.

2. **States where an excitation from a doubly occupied level occurs simultaneously with ionization**: This leads to states with 3 unpaired electrons, a class of excited states that would not be possible from photoionization, so does not have widely accepted nomenclature. The lowest energy states of this nature will be excitations from another Cl 3p lone pair level. Our probe cannot directly detect excitations into Cl 3p holes, as discussed in the main text. Therefore, signals from such states will not be immediately apparent. Indeed, such states could possibly lead to Cl^+, as noted later. One key observation is that these states cannot directly evolve to ground state CCl_3^++Cl, as the latter has only one unpaired electron. A state crossing that pairs two of the unpaired electrons could permit this, but preliminary computational investigations failed to reveal any such crossing. An alternative possibility is formation of excited state CCl_3^+ or Cl. The observed atomic Cl signals agree well with previous experimental results for ground state atomic Cl[24]. The S_1 and T_1 excited states of CCl_3^+ appear to absorb strongly at 285.7 eV from the C 1s → C 2p transition. No significant signal in that regime is however observed at the C K-edge, relative to the prominent ground state CCl_3^+ signal at 287.1 eV. Excited state CCl_3^+ formation therefore appears to not be particularly relevant.

   We also note that excitation of an electron from the bonding t_2 or a_1 levels (instead of the higher energy Cl 3p lone pairs) would lead to bright low energy spectral features similar to the ones predicted for the C/D states of the cation (from the core → hole transitions). Similar behavior can be expected for an excitation from the Cl 3s lone pair levels as well. Absence of such signals indicates the lack of such states.

3. **The unpaired electron of the ion is excited to a higher energy level:** This should be higher in energy than the previous type (for an excitation from a doubly

occupied 3p lone pair) and might be expected to also lead to Cl^+ formation, albeit with the atomic cation likely in a closed-shell singlet excited state.

4. **Double Ionization of CCl_4:** The recollision processes may have sufficient energy to not merely excite an electron in $CCl_4{}^+$, but to ionize it further to $CCl_4{}^{2+}$, as has been observed for other species[12,13]. The vertical double ionization energy of CCl_4 leading to the ground state of $CCl_4{}^{2+}$ is 28 eV, indicating that this is a potentially plausible outcome. Ground state $CCl_4{}^{2+}$ is a triplet, with both holes in the Cl 3p lone pair levels. The equilibrium geometry of this state is a D_{2d} symmetry form with a 1.75 Å bond length (vs 1.77 Å in neutral CCl_4). The computed C K-edge absorption features of this species are very similar to neutral CCl_4 (as the holes are on nonbonding Cl levels and bond lengths are similar), indicating that it will be invisible to this probe. It is thermodynamically favorable (by 4 eV) for this D_{2d} form of $CCl_4{}^{2+}$ to dissociate into $CCl_3{}^+$ and Cl^+. However, the barrier for this process is fairly large (0.70 eV) relative to the barrier for the C_{2v} form of $CCl_4{}^+$ dissociating into $CCl_3{}^+$ and Cl (0.17 eV). The lifetime of ground state $CCl_4{}^{2+}$ should thus be much larger than the 90 fs lifetime observed for the SBCB forms of $CCl_4{}^+$. It therefore appears rather unlikely that any dication (if formed) contributes significantly to the observed C K-edge transient signal at < 300 fs, as such species are likely to remain in the metastable D_{2d} form, which is invisible to our probe. Excited dication states involving holes in the bonding or Cl 3s lone pair levels are unlikely, as no bright low energy spectral features (similar to C/D photoionization states of the cation) are detected.

To summarize, excited states of $CCl_4{}^+$ and even doubly ionized $CCl_4{}^{2+}$ might reasonably be expected to form under experimental conditions. However, we find no evidence of species with holes in the σ_{CCl} bonding levels or Cl 3s lone pairs, which would lead to prominent low energy features in the transient spectra. Species with holes in the Cl 3p lone pairs are plausible as excitations that fill such holes are dark with respect to our probe (especially at the C K-edge). However, the observed features for the Cl formation channel appear to be adequately explained via just the X electronic state of $CCl_4{}^+$ alone, without need for any higher energy states. Higher vibrational states are however possible, though, and indeed quite likely. The potential role of excited $CCl_4{}^+$ states (with Cl 3p lone pair holes) in forming Cl^+ via a side channel is briefly discussed later.

V. ELECTRIC FIELD EFFECTS NEAR TIME ZERO

The presence of the pump electric field can cause additional signals in the ΔOD data during the temporal overlap of pump and probe. The most common effect in strong-field experiments is a mixing of electronic states with dipoles along the electric field, referred to as the Stark effect[25]. A peak pulse intensity of 3×10^{14} W/cm^2 corresponds to a root-mean-squared electric field strength of 0.05 atomic units (a.u.), which is large enough to significantly affect the spectrum. In general, the applied field should break the triple degeneracy of the $8t_2^*$ level and lead to mixing with the $7a_1^*$ level. Calculations of neutral CCl_4 with an applied field of 0.05 a.u. (at various orientations relative to the molecular axis) indicate that excitations to some of the initial $8t_2^*$ orbitals are blue-shifted in energy to $\sim$ 291.5 eV, while transitions to the former $7a_1^*$ level are around 288.2-288.6 eV (which is now weakly absorbing due to mixing with the $8t_2^*$). Thus, the positive feature at 288.8 eV in the experimental spectrum likely arises from neutral CCl_4 with antibonding levels perturbed by the electric field. This peak is observed in all pump intensity conditions, with larger shifts at higher intensities and smaller shifts moving away from time zero. The Stark effect also affects the spectrum of the ions, but the unionized species is present in greater amounts and hence is likely dominant in the observed spectrum.

VI. ADDITIONAL CHANNEL LEADING TO Cl^+ FORMATION

While the observed dissociation process to CCl_3^+ and Cl can be well explained by the intermediate forms and pathways discussed in the main manuscript, another channel that appears to result in neutral CCl_3 and cationic Cl^+ constitutes a significant part of the data. This channel is clearest at the chlorine $L_{2,3}$-edge where very sharp lines with similar widths to the atomic Cl lines are observed at 214 eV with less obvious components at 212.6 eV, shown in Fig. 3, which is the range that our ab initio calculations predict for the Rydberg states ($2p \rightarrow 3d, 4s$) of Cl^+. These atomically sharp peaks begin to appear with a delay of 37±6 fs relative to the onset of the main cationic CCl_4 signal, and from that point, it shows a time to rise of 85±10 fs for the best signal-to-noise dataset. For the intensity scan, these dissociation times are found to be 70±40 fs, 94±94 fs, and 229±559 fs, for 2.8, 2.3, and 1.8 $\times 10^{14}$ W/cm^2, respectively. This suggests the dissociation times of Cl^+ have a

slight dependence on pump intensity; although, uncertainties in the data do not allow a quantitative analysis. The delay of 37 fs is similar to the 23 fs delay of the 287.1 eV NBC signal to start appearing, which suggests that the time it takes for a Cl^+ atom to move far enough from the molecule to show atomic lines is about that time ∼37 fs, and that the nuclear motion may be similar to the case that forms neutral Cl.

After this delay, there is the somewhat long exponential time of 85±10 fs for the lines to reach their maximum value, which may suggest the presence of a shallow energy minimum, possibly corresponding to a NBC complex, before complete dissociation. The Cl^+ lines appear at much earlier times than Cl lines for two possible reasons. Firstly, the 3d and 4s levels of the cation have greater valence character due to the positive charge, leading to stronger absorption that is harder to broaden into the baseline. Secondly, the associated CCl_3 moiety for this pathway is neutral and thus plays less of a role in influencing the energy

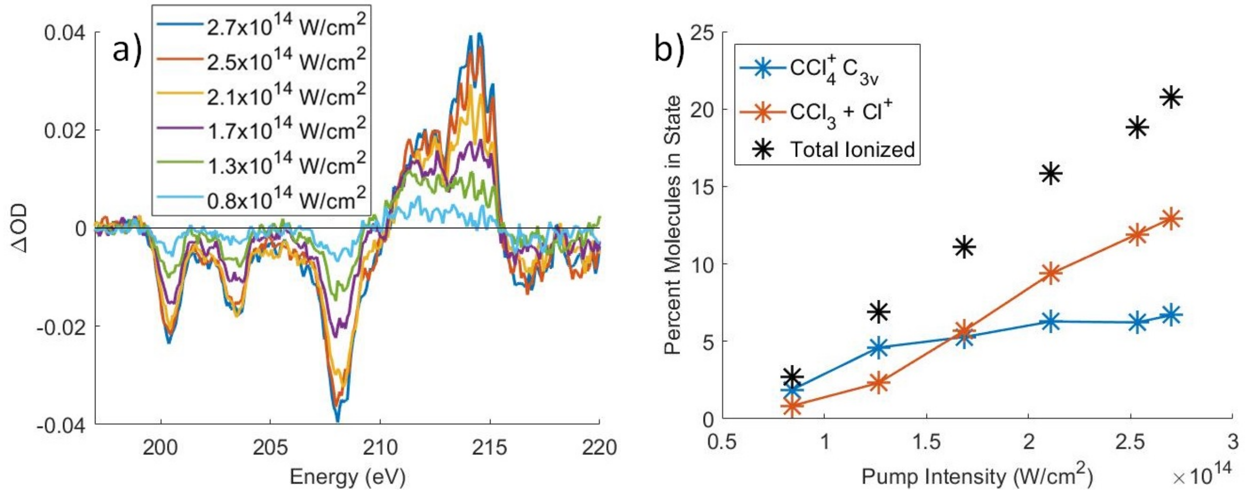

FIG. 3. a: Spectral lineouts averaged from 190-230 fs as a function of increasing pump power, measured before the vacuum chambers and recombination mirror. Note that at high powers, the sharp lines at 214 eV corresponding to Cl^+ become increasingly prevalent. b: Percentage of molecules in each channel between generation of atomic neutral Cl or cationic Cl^+. The X-axis uses estimated intensities of the pump pulse, but the relative intensities are accurate. At the powers and pulse duration used, the amount of the neutral channel plateaus quickly and has only slight increases with increasing power, whereas the Cl^+ channel increases nearly linearly after $\sim 1.3\times10^{14}$ W/cm^2. At the highest power, the relative amounts of Cl and Cl^+ from $CCl_4{}^+$ dissociation are 35% and 65%, respectively.

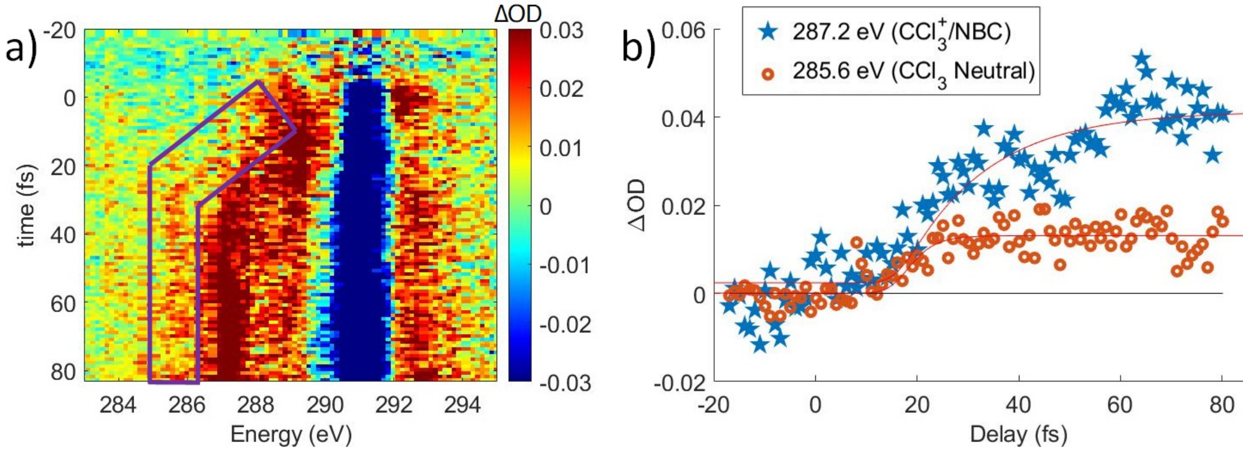

FIG. 4. a: ΔOD colormap at the C K-edge with colors saturated to better show a feature that splits from the main feature and continues to 285.5 eV. b: Lineouts comparing the growth of the 287.2 eV $CCl_3{}^+$/NBC feature to the 285.5 eV feature, which is assigned to neutral CCl_3. The NBC lineout has a delay of 23 ± 8 fs and a rise time of 50 ± 20 fs. The CCl_3 lineout has a delay of 20 ± 10 fs and a rise time of 8 ± 6 fs.

levels of Cl^+ than $CCl_3{}^+$ does for Cl.

At the C K-edge, evidence of this channel can also be seen in a feature that appears at 285.6 eV, shown in figure 4. OO-DFT calculations show CCl_3 is expected to have absorption at 285.5 eV. It shows a delay in appearance of 20 ± 10 fs, followed by a rise time of 8 ± 6 fs. The delay is very similar to the 23 ± 8 fs delay in the NBC formation, which was associated with the time required for the nuclear motion to break the covalent bond for the molecules that dissociated immediately. Again the similarity of the delay suggests similar nuclear motion along the pathways. However, the much shorter rise time suggests that only the molecules that dissociate immediately contribute to the Cl^+ formation pathway.

The formation of Cl^+ is a much higher energy channel, with a final energy that is 4.8 eV higher than the Cl dissociation channel. Cl^+ formation has been observed in previous CCl_4 ionization experiments from both electron impact[26] and single-photon ionization[27,28]. However, it was always found to be a minor channel, with an abundance of 0-5% around ∼ 20 eV ionization energy. A larger percentage of Cl^+, ∼36%, is observed in strong-field ionization[29], and, in the power scaling shown in Fig. 3, we observe more than 65% forming, with the difference from other results[29] likely arising from shorter pulse durations and higher intensities. The high energy of this channel appears to suggest that it arises via an

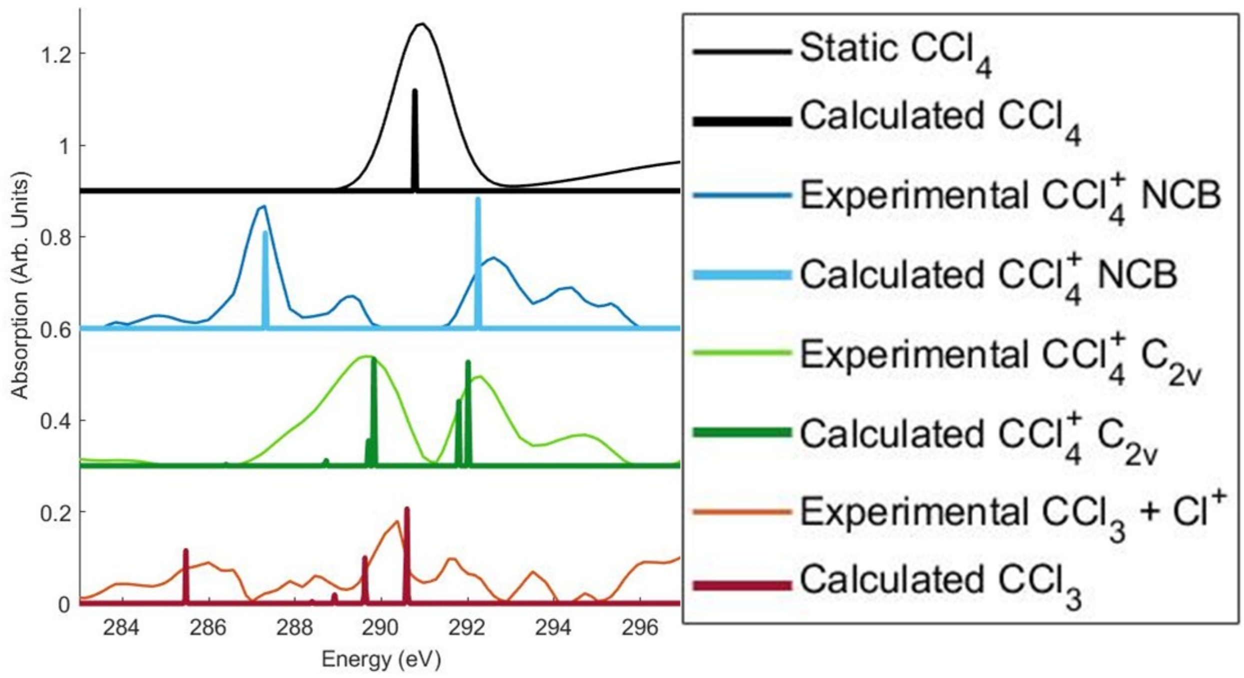

FIG. 5. A comparison between experiment and theory, including neutral CCl_3, SBCB form, and NBC shows good agreement with each.

excited state, potentially one involving an electron excited from the Cl lone pair SALCs to the anti-bonding σ^*_{CCl} orbitals. Such a state would not be acessible under single-photon photoionization conditions. Occupation of a repulsive σ^*_{CCl} level in particular, can lead to rapid bond cleavage.

VII. TIME CONSTANT CHANGES WITH VARYING PUMP INTENSITY

In order to determine the dependence of the lifetimes measured on the pump intensity, a measurement was taken that varied the pump power. The parameters of the power scan are as follows: For the part near time zero, 2.5 fs steps are used, out to 40 fs. Then 60 fs steps are used to 400 fs. Powers used between these are directly comparable to each other, as the data was taken as simultaneously as possible. A scan from -20 to 400 fs (~6 min process was taken at one power, followed by a scan at the next power, and so on until a full scan was taken at each power. Then, another scan at the first power was taken, followed by the next, and so on for ~8 hours total. The power dependence of signals were determined from this power scan. These data do not show a statistically significant difference in the $T_d \rightarrow$ SBCB

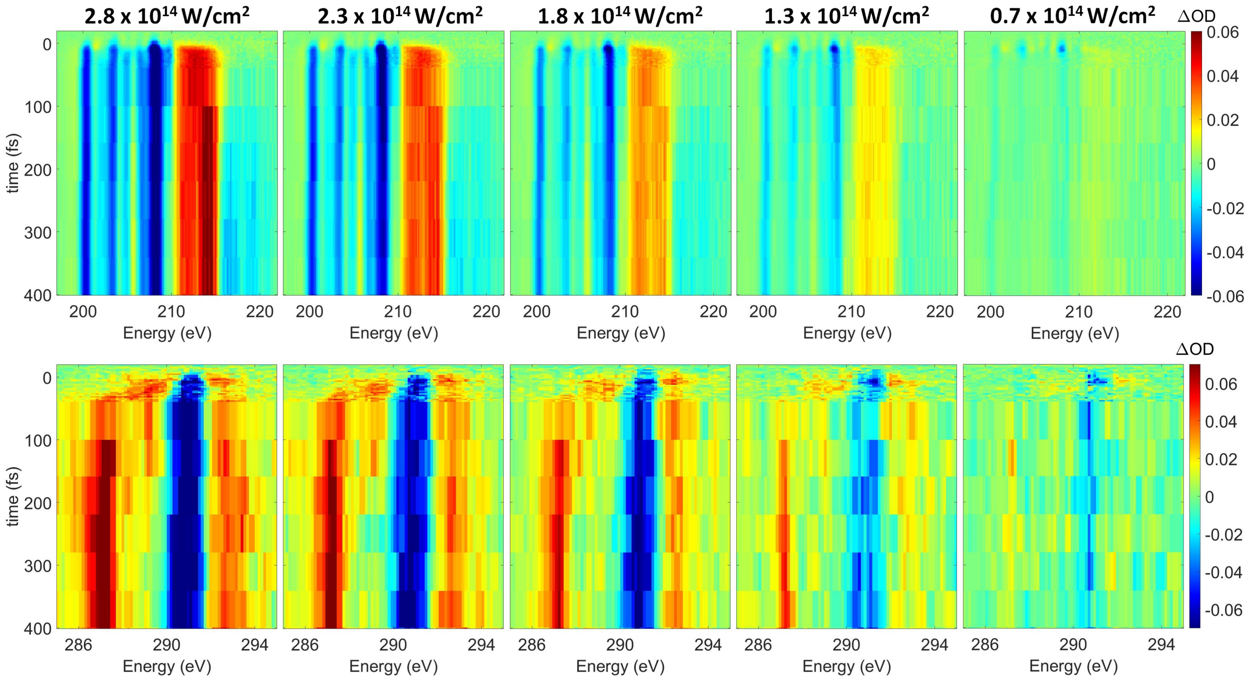

FIG. 6. Experimental data taken as a power scan. Top: Cl L-edge. Bottom: C K-edge. The estimated intensities of the pump pulse in each condition is shown. Extracted lifetimes from fitting are given in Table I.

Estimated Intensity	$T_d \rightarrow$ SBCB (fs)	SBCB $\rightarrow$ NBC (fs)	Atomic Cl^+ rise at 214 eV (fs)
2.8×10^{14} W/cm^2	8 ± 4	90 ± 10	70 ± 40
2.3×10^{14} W/cm^2	5 ± 4	90 ± 10	90 ± 90
1.8×10^{14} W/cm^2	4 ± 5	90 ± 20	200 ± 600
1.3×10^{14} W/cm^2	6 ± 6	100 ± 20	None observed
0.7×10^{14} W/cm^2	6 ± 6	90 ± 50	None observed

TABLE I. Time constants for fits of the data in Fig. 6. Powers are shown as estimated intensity. The atomic Cl^+ rise time refers to the sharp spectral features at 214 eV and each rise follows a ~37 fs delay, as described in section VI.

time or the SBCB $\rightarrow$ NBC time. The scan shows a possible difference in the lone, ionic Cl^+ formation time. The relative amounts of Cl^+ and Cl formation are also determined from this scan. The ΔOD data are shown in Fig. 6, and the fitted lifetimes are given in Table I.

VIII. CALCULATED GEOMETRIES AND ENERGIES OF INTERMEDIATES

The exact structures optimized and used for calculations are given for each species as separate xyz files, with key structural parameters noted in Table II. Structures were optimized with the ωB97M-V[30] density functional and the aug-pcseg-3[31] basis set. Zero-point energies were found at the same level of theory. Relative ground state electronic energies at the optimized geometries were computed with CCSD(T)[32] extrapolated to the complete basis set (CBS) limit. The CBS extrapolation was done as follows:

$$E\,[\text{CCSD(T)/CBS}] = E\,[\text{HF/CBS}] + E_{corr}\,[\text{CCSD(T)/CBS}] \tag{12}$$

$$E\,[\text{HF/CBS}] \approx E\,[\text{HF/aug-cc-pV5Z}] \tag{13}$$

$$\begin{aligned} E_{corr}\,[\text{CCSD(T)/CBS}] \approx & \frac{64E_{corr}\,[\text{fc-CCSD(T)/aug-cc-pVQZ}] - 27E_{corr}\,[\text{fc-CCSD(T)/aug-cc-pVTZ}]}{64-27} \\ & + E_{corr}\,[\text{CCSD(T)/aug-cc-pCVTZ}] - E_{corr}\,[\text{fc-CCSD(T)/aug-cc-pCVTZ}] \end{aligned} \tag{14}$$

where E_{corr} is the correlation energy, and fc stands for frozen-core (i.e. only correlation from valence electrons is computed). The frozen-core correlation energy is extrapolated to the complete basis set limit using the two point, cubic extrapolation formula from Ref 33. The Dunning aug-cc-pVnZ (n=T,Q,5) basis sets[34–36] were used for this purpose. Correlation arising from core-electrons is corrected for via the $E_{corr}\,[\text{CCSD(T)/aug-cc-pCVTZ}] - E_{corr}\,[\text{fc-CCSD(T)/aug-cc-pCVTZ}]$ term, which is the difference between all-electron and frozen-core CCSD(T) results for the aug-cc-pCVTZ basis set[37,38].

	Energy (eV)	r_{C-Cl_1} (Å)	r_{C-Cl_2} (Å)	$\measuredangle_{Cl_1,C,Cl_1}$ (°)	$\measuredangle_{Cl_1,C,Cl_2}$ (°)	$\measuredangle_{Cl_2,C,Cl_2}$ (°)
CCl_4^+ T_d	0.57	1.76		109.5		
CCl_4^+ C_{2v}	0.18	1.70	1.82	115.64	86.11	112.90
CCl_4^+ NBC C_{3v}	-0.15	1.64	3.38	119.90	90.43	
CCl_3^+ and Cl	0	1.64		120		
CCl_4	-11.13	1.77		109.5		

TABLE II. The calculated CCSD(T)/CBS energies, bond distances, and angles are given for the intermediates identified in this experiment. The dissociation limit energy is set to 0. For species with two inequivalent types of chlorines, Cl_1 always refers to the one with the shortest bond distance from C. For fully equivalent species, the Cl_2 terms are omitted.

IX. CALCULATED NORMAL MODES

The normal modes were found at the ωB97M-V/aug-pcseg-3 level of theory. Lack of rotational symmetry due to the finite nature of the numerical grid used for evaluating DFT exchange-correlation integrals lead to slight differences between computed normal mode frequencies that should be degenerate in theory. For instance the asymmetric stretch of CCl_4 is triply degenerate, but the computed frequencies range from $810.24 - 811.49$ cm^{-1}.

	CCl_4		Covalently bonded CCl_4^+	
Experiment[39]	Theory	Period	Theory	Period
(in cm^{-1})	(in cm^{-1})	(in fs)	(in cm^{-1})	(in fs)
217	220.45	151.31	198.92	167.69
	220.50	151.28	236.59	140.99
314	319.29	104.47	292.70	113.96
	319.31	104.46	299.41	111.41
	319.42	104.43	329.51	101.23
459	476.19	70.05	482.55	69.13
776	810.24	41.17	534.72	62.38
	811.02	41.13	801.70	41.61
	811.49	41.11	926.68	36.00

TABLE III. Normal mode frequencies of CCl_4 and covalently bonded $CCl_4{}^+$ in cm^{-1}. The oscillation period (in fs) computed from theoretical frequencies is also reported. Experimental normal mode frequencies of neutral CCl_4 are provided for comparison.

X. CALCULATED C K-EDGE TRANSITIONS AND ASSIGNMENTS

OO-DFT[40]/SCAN[41] calculated energies of transitions and their assignments from the theoretical calculations are given for each species. Detailed protocols for running such calculations are described in Refs 42 and 43. Excited state orbital optimization was done with the square gradient minimization (SGM[44]) and initial maximum overlap method (IMOM[45]) algorithms, for restricted open-shell and unrestricted calculations, respectively. The aug-pcX-2 basis[46] was used at the site of the core-excitation, and aug-pcseg-2[31] was used for all other atoms. Energies are not empirically shifted to align with experiment.

Neutral CCl_4		
Transition Energy (in eV)	Oscillator Strength	Assignment
289.00	6.5796E-08	$7a_1^*$ (C -Cl σ^*)
290.77	0.07267	$8t_2^*$ (C -Cl σ^*)
290.77	0.07267	$8t_2^*$ (C -Cl σ^*)
290.77	0.07267	$8t_2^*$ (C -Cl σ^*)

TABLE IV. C K-edge energies for neutral CCl_4 used in Fig 5. The C $1s \rightarrow 7a_1^*$ transition is forbidden, and does not appear in the experimental spectrum. The $8t_2^*$ level is triply degenerate, leading to three transitions (as shown).

$CCl_4{}^+$ C_{2v}		
Transition Energy (in eV)	Oscillator Strength	Assignment
286.4	0.001031	SOMO (Cl 2p)
288.6474	0.000738	C -Cl symmetric σ^* (long bonds)
289.7012	0.018316	C -Cl symmetric σ^* (long bonds)
288.7216	0.003823	C -Cl antisymmetric σ^* (long bonds)
289.8263	0.077694	C -Cl antisymmetric σ^* (long bonds)
290.573	0.000134	C -Cl symmetric σ^* (short bonds)
291.7935	0.046993	C -Cl symmetric σ^* (short bonds)
290.8488	1.21E-07	C -Cl antisymmetric σ^* (short bonds)
292.0107	0.075429	C -Cl antisymmetric σ^* (short bonds)

TABLE V. C K-edge energies for the C_{2v} form of $CCl_4{}^+$ used in Fig 5. The C_{2v} form has 2 inequivalent bond types with different characteristic σ^* energies. The transition from the carbon 1s to the chlorine 2p SOMO is computed to be at 286.4 eV, but with very low intensity, and this transition is not observed in the experimental data.

CCl_4^+ NBC / CCl_3^+		
Transition Energy (eV)	Oscillator Strength	Assignment
291.07	4.40E-05	SOMO (Cl 2p hence CT)
286.0362	7.71E-09	C 2p
287.3108	0.07711	C 2p
289.5408	4.24E-08	C-Cl σ^* (a_1)
290.8483	1.57E-05	C-Cl σ^* (a_1)
291.0506	1.51E-05	C-Cl σ^* (E)
292.2435	0.064844	C-Cl σ^* (E)
291.069	1.50E-05	C-Cl σ^* (E)
292.2341	0.06492	C-Cl σ^* (E)

TABLE VI. The transition energies from the carbon 1s for the global minimum NBC form of CCl_4^+ used in Fig 5.

CCl_3		
Transition Energy (eV)	Oscillator Strength	Assignment
285.47	0.038337	SOMO (C2p like)
288.3928	0.001316	C-Cl σ^* (a_1)
288.9165	0.006173	C-Cl σ^* (a_1)
289.6242	0.023365	C-Cl σ^* (E)
290.5987	0.049872	C-Cl σ^* (E)
289.6126	0.023154	C-Cl σ^* (E)
290.5866	0.048831	C-Cl σ^* (E)
291.4134	0.000343	Rydberg a_1
292.7127	0.000317	Rydberg a_1

TABLE VII. The transition energies from the carbon 1s for neutral CCl_3 used in Fig 5.

XI. COMMENT ON QUASICLASSICAL TRAJECTORY CALCULATIONS

We had utilized quasiclassical trajectory (QCT) calculations[47] to model the short-time symmetry breaking of T_d CCl_4^+. These calculations assume nuclei to be classical particles and propagate them on the ground state electronic potential energy surface produced from ωB97M-V/aug-pcseg-1. The calculations therefore are on a single electronic energy surface (i.e. are adiabatic). The ionization process is assumed to be Franck-Condon, with the cation initially being in the neutral CCl_4 equilibrium geometry at $t = 0$. All 9 normal modes of the molecule are given kinetic energy corresponding to the zero-point energy (ZPE) of neutral CCl_4. Each normal mode has two possible velocity directions, leading to $2^9 = 512$ possible trajectories. The pump pulse likely supplies even greater amounts of energy into the vibrational modes, but this is not explored. Indeed, all the trajectory calculations were run in the absence of the field, to provide only a zeroth-order picture of the short time dynamics of the system. Absence of the field effects in fact might slow down the rate of processes in QCT vs experiment.

The QCT trajectories however are not particularly reliable at long times due to the well-known problem of ZPE leakage[48]. The nuclear motion is classical, and there is no guarantee that each mode continues to retain at least ZPE of energy. Indeed, a 'leakage' is generally observed from high frequency to low frequency modes over time, affecting the dynamics. Such ZPE leakage affects our QCT simulations in two ways. Firstly, formation of the non-covalently bonded complex from covalently bonded CCl_4^+ involves an asymmetric stretch in the latter, which has relatively high frequency compared to the bending modes. Energy leakage out of the stretching modes to lower frequency modes therefore would hinder non-covalent complex formation, leading to the covalently bonded form persisting longer in the QCT calculations. Secondly, the noncovalent complex forms have three very low frequency modes corresponding to motion of atomic Cl relative to the CCl_3^+ moiety, and spurious energy flow into these modes through ZPE leakage would lead to rapid dissociation of the complex into constituent fragments. The ZPE leakage problem in the QCT calculations therefore appears to disadvantage the noncovalent complex, both by slowing the formation rate as well as reducing the lifetime against dissociation. The long time behavior of QCT trajectories therefore are not likely to be reflective of the actual dynamics of the system.

XII. FREQUENCY OF MOLECULE-MOLECULE AND MOLECULE-ION COLLISIONS

The CCl_4 was purchased as a liquid and was made gaseous by exposure to the vacuum, using a needle valve to regulate the pressure. The gas pressure of the CCl_4 in the foreline was set to 12 mbar. No heating elements were used, so the temperature should be less than or equal to room temperature in the sample cell. Therefore, the number density of CCl_4 molecules is:

$$\frac{N}{V} = \frac{p}{k_B T} = 2.9 \times 10^{23} m^{-3} \tag{15}$$

$$\tag{16}$$

Collision theory yields the frequency (per unit volume) of molecule-molecule collisions to be:

$$Z = (\pi R^2) v_{rel} \left(\frac{N}{V}\right)^2 \tag{17}$$

where πR^2 is the cross-sectional area of the collision and v_{rel} is the average relative velocity between particles. The 'lifetime' of a single molecule against collision with another molecule is then given by:

$$\tau_{MM} = \frac{1}{(\pi R^2) v_{rel} \left(\frac{N}{V}\right)} \tag{18}$$

From kinetic theory:

$$v_{rel} = \sqrt{\frac{8\pi k_B T}{\pi \mu}} \tag{19}$$

where μ is the relative mass for the colliding particles. For CCl_4 $\mu = \frac{151.8754}{2}$ a.m.u. or 6.3×10^{-26} kg. Consequently, $v_{rel} = 408$ m/s. The cross-section radius R is estimated to be twice the C-Cl bond-length (1.76 Å) plus twice the van dar Waals radius of Cl (2 Å). With this, we obtain a cross-sectional area of 1.8×10^{-18} m^2. All of these quantities collectively yield $\tau_{MM} = 4.7$ ns. This indicates that collisions between molecular species in our experiment occurs in the timescale of nanoseconds, and is thus not relevant on the timescales observed here. All the studied processes can therefore be safely assumed to be unimolecular.

A more challenging case is the possibility of collisions between molecular species and the electrons produced by strong-field ionization. Such electrons can be much faster, permitting more frequent collisions that might be of comparable timescales as the observed dynamics. We therefore attempt to find a lower bound for such collision lifetimes to determine if they are relevant. Since only about a quarter of the molecules are ionized at most (from Fig 3), the electron density is at most $\frac{N}{4V}$. If we assume the electrons have an energy of 20 eV, then they will have a speed of $v_{elec} = 2.6 \times 10^6$ m/s. This is much larger than the thermal velocity of molecular species, and thus we can assume $v_{rel} \approx v_{elec}$ for such collisions. Literature estimates for electron-CCl_4 collision cross-section is at most $A = 10^{-18}$ m^2 for such electron energies[49]. With this, we obtain the mean lifetime of a molecular species for electron collision to be $\tau_{Me} = \frac{1}{Av_{elec}\left(\frac{N}{4V}\right)} = 5.2$ ps. This is close to the upper limits of the greatest timescales measured in the experiment, but is large enough to indicate that molecule-electron collisions are unlikely to be relevant for any process other than the slow dissociation of the NBC to atomic Cl. Even that process is considerably faster (800 fs) at high pump powers, such that collision with ionized electrons should be irrelevant. Collisions with electrons may become relevant at lower pump powers where the NBC lifetime is longer, but it is worth noting that the ionized electron density will be even lower at those powers, leading to even less frequent collisions. We note that the very fast electrons that are most likely to cause collisions are also the ones that would be least affected by the electrostatic field of any cationic molecular species, indicating that the provided estimate for collision time is adequate even though it is based on neutral CCl_4.

XIII. SEPARATION OF SPECTRAL COMPONENTS FROM EXPERIMENTAL DATA BY MULTIVARIATE FITTING

After the data for each dataset is prepared and saved in files containing the ΔOD for the chlorine and carbon edges, the static absorption generated by the pump-off spectra and a no gas spectrum from the start of the run, delay axis, and pixel-to-energy calibration axis. A dataset is the set of data taken continuously with no changes to the experiment, usually divided by being from different days, but also possible single days with multiple conditions. Slight differences in energy calibration and sample pressure are determined by

comparing the static absorption of each dataset to a master static spectrum. Changes are made accordingly to make each dataset comparable to each other. These are all loaded into a single program to fit all of them to a model. An example of a finished fit for one dataset is shown in Fig. 7.

The model follows the general assumption that

$$\Delta OD = (P_1 \times OD_1 + P_2 \times OD_2 + P_3 \times OD_3 + \dots + P_{static} \times OD_{static}) - OD_{static}$$

where, $P_1 + P_2 + P_3 + \dots + P_{static} = 1$ are coefficients denoting the percent of molecules in a particular state at a particular time and P_{static} is the percent that remain in neutral CCl_4. The optical density, OD, of each of the states is the goal of this fitting procedure. The number of states involved is able to be changed, based on the expected number of states. In this case, at least 3 are necessary to get a decent representation of the data, the C_{2v}, C_{3v}, and CCl_3 + Cl^+ forms.

The new absorptions, OD_1, OD_2, etc., are represented as parameters simply by absorp-

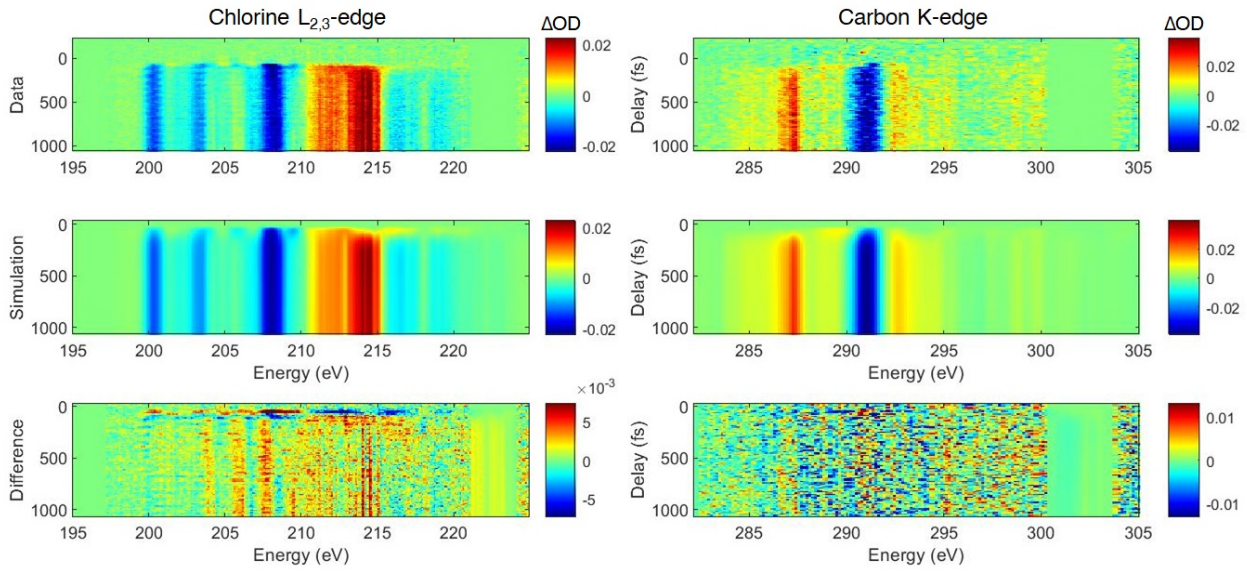

FIG. 7. An example of one dataset fitted by the multivariate fitting algorithm. Both the chlorine and carbon edges are shown in the two columns. The top row shows the data. The middle row shows the result of the fitting. The bottom row shows the difference between the data and the fitting (the top and middle rows). Note that the colorscale on the bottom row is zoomed by a factor of 2 to highlight errors. This shows only one dataset. For this fitting, 9 datasets are fitted simultaneously. The others are not shown for space considerations.

tions spaced linearly between 2 energies. For example, the Cl L-edge used 201 points, and the C K-edge 81 points. These linear spacings were interpolated onto the nonlinear energy axis of each dataset. This representation of absorptions is very inefficient in terms of numbers of parameters, but it is exceptionally faster in converging to the global minimum. A set of pairs of absorption and the energy spacing from the previous point was also tried that needed about 1/3 of the number of parameters, but it took ∼40 times as many iterations to converge. The more chemically accurate representation with Gaussians corresponding to particular transitions was also attempted, and while it could represent the spectra with the fewest parameters, 30 for the Cl L-edge and 15 for the C K-edge, the parameter space was so riddled with local minima that it often would not change the parameters from their initial values.

The states are evolved in time to allow one state to become another and capture the dy-

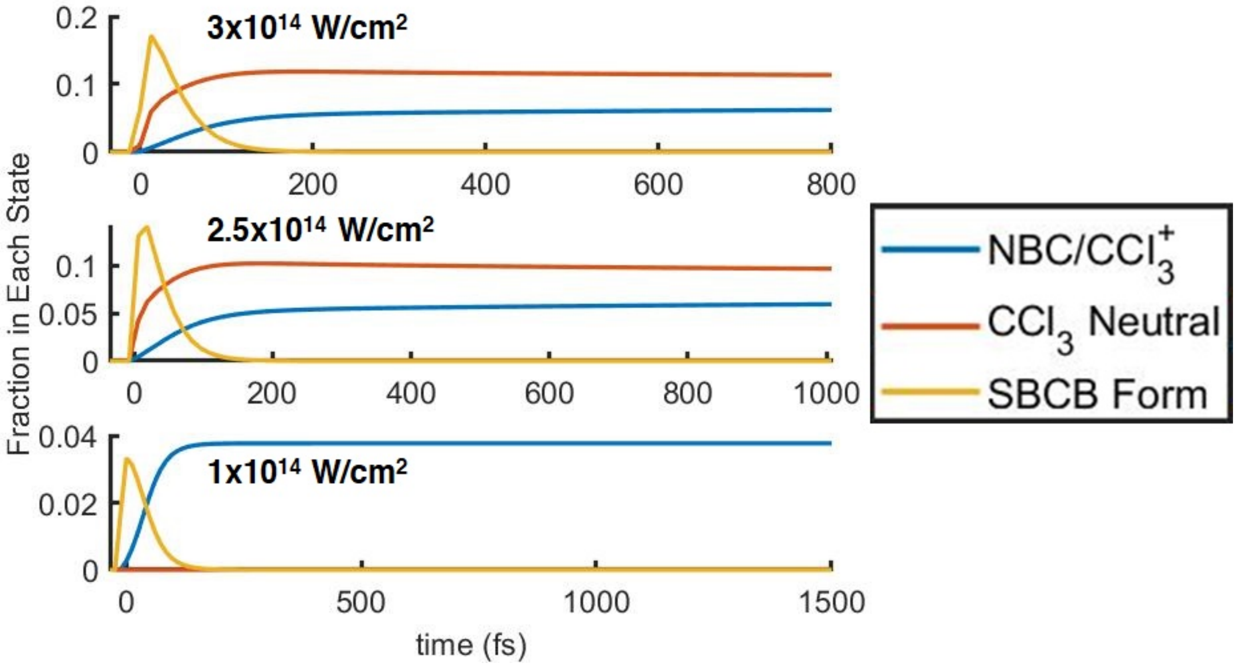

FIG. 8. An example of the population curves used in a fitting are shown for 3 datasets in a 9 dataset fitting. The fraction in each state is defined such that 100% = 1, 20% = 0.2, etc. The 3 datasets represented have approximate intensity of the pump pulse of 3×10^{14} W/cm^2, 2.5×10^{14} W/cm^2, and 1×10^{14} W/cm^2. The colors of the curves represent the same states in each plots, blue: NBC, red: CCl_3 neutral after Cl^+ dissociates, and yellow: SBCB form.

namics of the system. When one state becomes another, its population is simply transferred into the absorption of the new one with no intermediate transition, because doing so would overly complicate the algorithm with minimal gain. The method of population fitting is to represent each state's P population as a function of time is with two single exponentials for rise and decay:

$$P_1(time) = (erf((x-c)/0.1fs)+1) \times (-1*a*e^{(x-c)./(-1*\tau_{in})}+a) \times ((1-d)*e^{(x-c)/(-1*\tau_{out})}+d)$$

where c is a time offset to compensate for poorly decided time zeros, τ_{in} is the time constant for rise, τ_{out} is that for decay, a determines the maximum population, and d determines the final population of that state as a percentage of the maximum. An example of population curves resulting from a fit are shown in Fig. 8. The error function, erf, at the beginning is to set the population to zero before ionization and allow it to evolve after that. This function is chosen over subtraction of exponentials in order to be able to allow a population to finish at an arbitrary value, rather than 0, so that the same function can be used for initial, intermediate, and final states. Population curves are created for each state, and the curves are then normalized to ensure $P_1 + P_2 + P_3 + ... + P_{static} = 1$ at each time point. This is done to reduce the amount of local minima in the parameter space so that the fit can better find the global minimum, and this is the reason for several design decisions in this program. A potentially more accurate model might have been to assign k values between each state and calculate the populations numerically as a function of time; however, this would have required more time, which is anathema to use with a fitting procedure.

The amount of total ionization for each dataset was determined by fitting to a single new state and minimizing the second derivative by transition energy of that state's absorption. This is similar to what is done in static spectral add back techniques[50,51], which will result in a percent ionization chosen that minimizes the contribution of the original static spectrum in the new spectra while ensuring that there is no negative absorption. When multiple new states are used, the ionization percent will tend towards 100% for the most intense cases, so it is instead held constant at the result determined by a single state. Minimization of the second derivative of the new states is also used when multiple new states are fitted to reduce overfitting between multiple states; however, it makes things like the Rydberg lines of Cl and Cl^+ get smoothed over.

In our data, we also see vibrations of ~455 cm^{-1}, which corresponds to the symmetric

stretch in neutral CCl_4, which will be further described in a later paper. These vibrations are taken into account in the program by moving the energies of the transitions to the molecular orbitals (σ^* $7a_1^*$ and σ^* $8t_2^*$). The static absorption is divided into Gaussians representing the transitions; and as a function of time, the centers of these Gaussians are moved in accordance with a sine function with the amount of movement dependent on the core-excited slope specific to that transition.

The ΔOD as a function of time is then calculated based on this model and is broadened by convolution with a Gaussian to account for the temporal resolution of our experiment.

The model is subtracted from the data and fed into a minimization of least squares fitting algorithm, lsqnonlin in Matlab. The parameters that are common to all datasets are:

1. OD of each new state
2. Frequency of the neutral vibration
3. Core-Excited state slopes for the vibration of each transition
4. The lifetime constants, τ_{in}, τ_{out}, and d, for each of the new states

The parameters that are unique to each individual dataset are:

1. Timing delay offset, c
2. Population parameters, a, for each state in each dataset
3. Vibrational amplitude of the neutral
4. Temporal broadening amounts

XIV. METHOD OF INTENSITY ESTIMATION BY Ar IONIZATION

A multiple measurements were taken at different powers on an Ar sample and the XUV features associated with Ar^+ were added together into negative and positive features for t 100 fs. The relationship between ΔOD and ionization rate should be proportional, so the signals were linearly scaled until good agreement was found with the Perelomov, Popov and Terent'ev's model (PPT) rate of the ionization of Ar[52] via a fit. Because of the linear scaling, the shape of the ionization rate and the power/intensity at which the rate changes

are the largest factors in finding the correct intensity. The fit gives a rough approximation, but it gives an order of magnitude estimate for the electric field intensity of the pump of $2.7 \pm 1.1 \times 10^{14}$ W/cm^2 at maximum power, shown in figure 9. This is of the same order of magnitude as the peak intensity, 5×10^{14} W/cm^2 given by calculation using 150 μJ, 6 fs FWHM duration, and 65 μm FWHM spot size. The calculation assumes that the pulse is perfectly Gaussian in time and space, which is not necessarily true, especially since the beam is made collinear with an annular mirror. The calculation is expected to be an overestimate of the intensity, which is what is observed with comparison to Ar ionization.

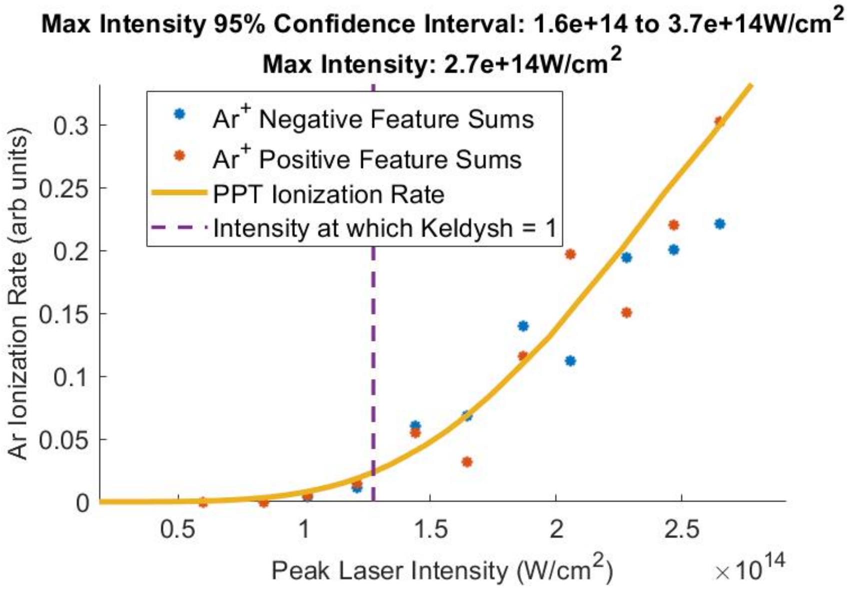

FIG. 9. Internal calibration of the pump electric field intensity is done by comparison to the ionization rate of argon, using the PPT rate[52]. The intensity for which the Keldysh parameter is equal to 1 is also indicated, which is the intensity above which ionization is generally considered to be tunnel ionization, compared to multiphoton ionization[53].

XV. FURTHER ANALYSIS OF DISTORTION FROM TRAJECTORY CALCULATIONS

To better visualize the distortions in the SBCB form, the average of each bond length is shown in Fig. 10. The lengths are binned together by their relative length at any given time. This ignores the particular identities of which chlorine the bond corresponds to and ensures that there will be no curve crossing. The averaging only includes ions that are still

in the SBCB form, those that have 4 covalent C-Cl bonds, the number of which is shown in the green line on the right y-axis. It shows that the two long bonds extend outward from the ground state after ionization as the two short bonds contract. Following this, vibration-like behavior is observed as the two long and two short bonds mirror each other.

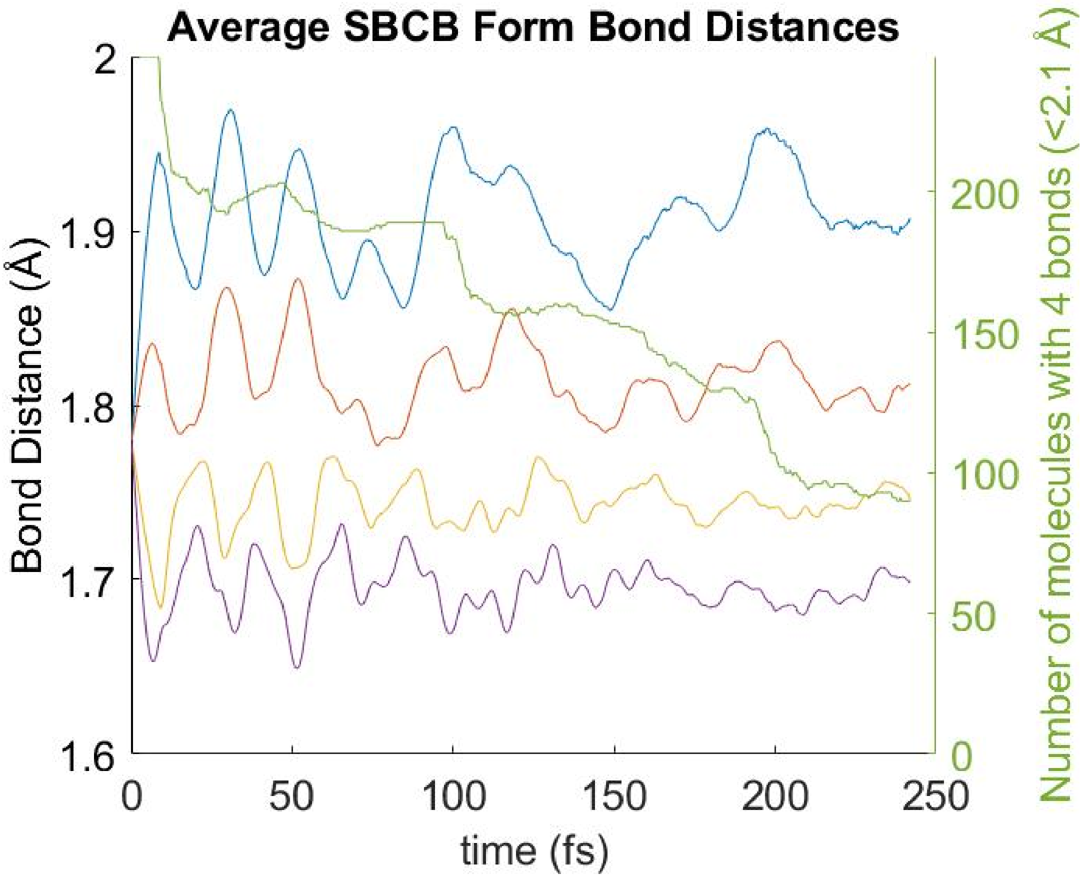

FIG. 10. Average bond lengths for the CCl_4^+ ions that remain in the SBCB form are shown. The longest two bonds show opposite behavior to the two shortest bonds, consistent with a C_{2v}-like structure.

In order to determine when the ion is expected to distort to the C_{2v} minimum of CCl_4^+, a variant of the root-mean-square deviation (RMSD) of atomic positions is used to compare the calculated positions at a given time from the trajectory calculations to the calculated C_{2v} minimum structure. The exact formula used is:

$$RMSD = \sqrt{\frac{1}{N_{C-Cl}} \sum_{i=1}^{N_{C-Cl}} \delta_i^2 + \frac{1}{N_{Cl-Cl}} \sum_{i=1}^{N_{Cl-Cl}} \delta_j^2}$$

where δ is the difference between the inter-atomic distances for the trajectory distance and

the C_{2v} distance. This internal coordinate is used instead of the external XYZ coordinate to eliminate rotations and translations. A lower number indicates a structure more similar C_{2v} structure. The initial value for the tetrahedral CCl_4 is 0.44, and the first minimum appears at 26.6 fs with a value of 0.09. The RMSD becomes large when one of the C-Cl bonds break.

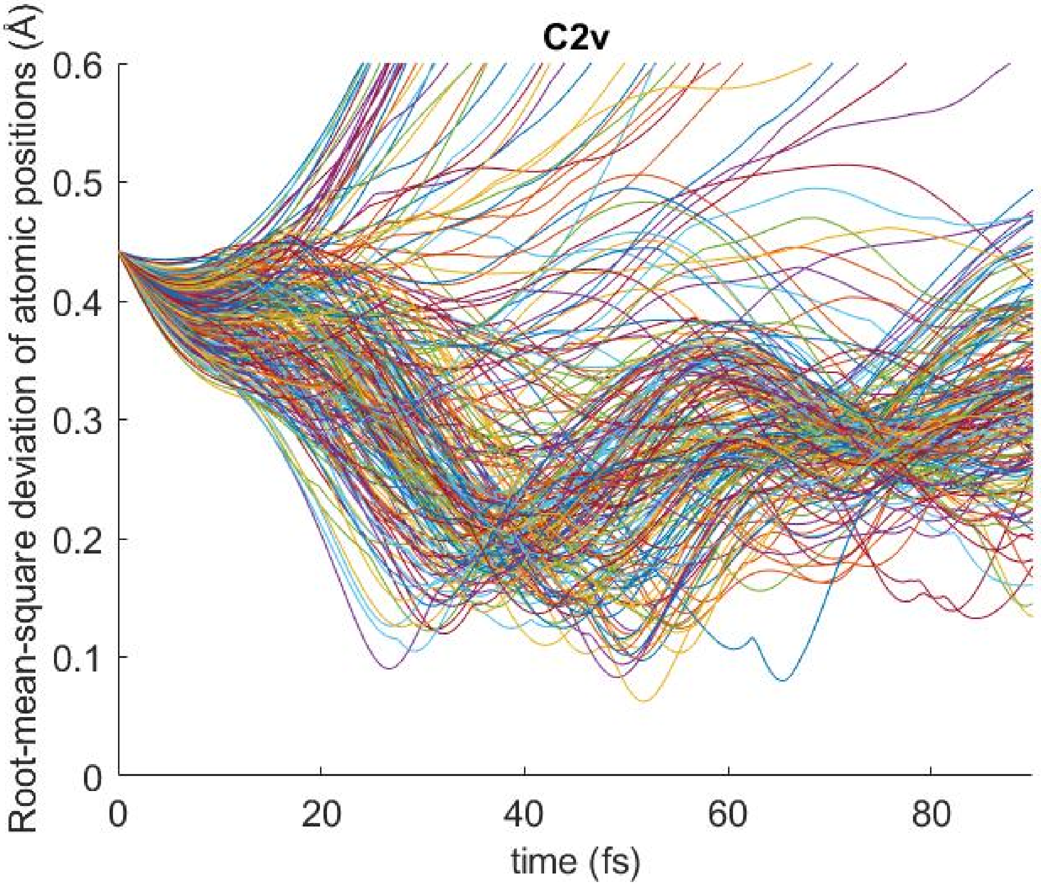

FIG. 11. Root-mean-square deviation of atomic positions from the local minimum C_{2v} for all trajectory calculations. The smaller the number, the closer the structure is to C_{2v}. Trajectories show the ion can become the C_{2v}-like as early as 26.6 fs.

XVI. ADDITIONAL CCl_4^+ DATA

In the main text, several lineouts and timescales are taken using multiple datasets. These additional datasets comprise too many colormaps to show effectively in the main text, so they are shown here for completeness. Also shown are lineouts taken at 2 different times from before and after the dissociation of the free Cl in Fig. 15. These lineouts show that

the changes in the spectra over the free Cl dissociation are minor, except in the area of 204-210 eV, where the atomic spectral features of the Cl become visible. Static and transient data at the Cl L_1-edge are shown in Fig. 16. The data for this figure come from the same measurement as the data for Fig. 2 in the main text. This edge was not analyzed due to the lower signal-to-noise ratio, as compared to the other edges.

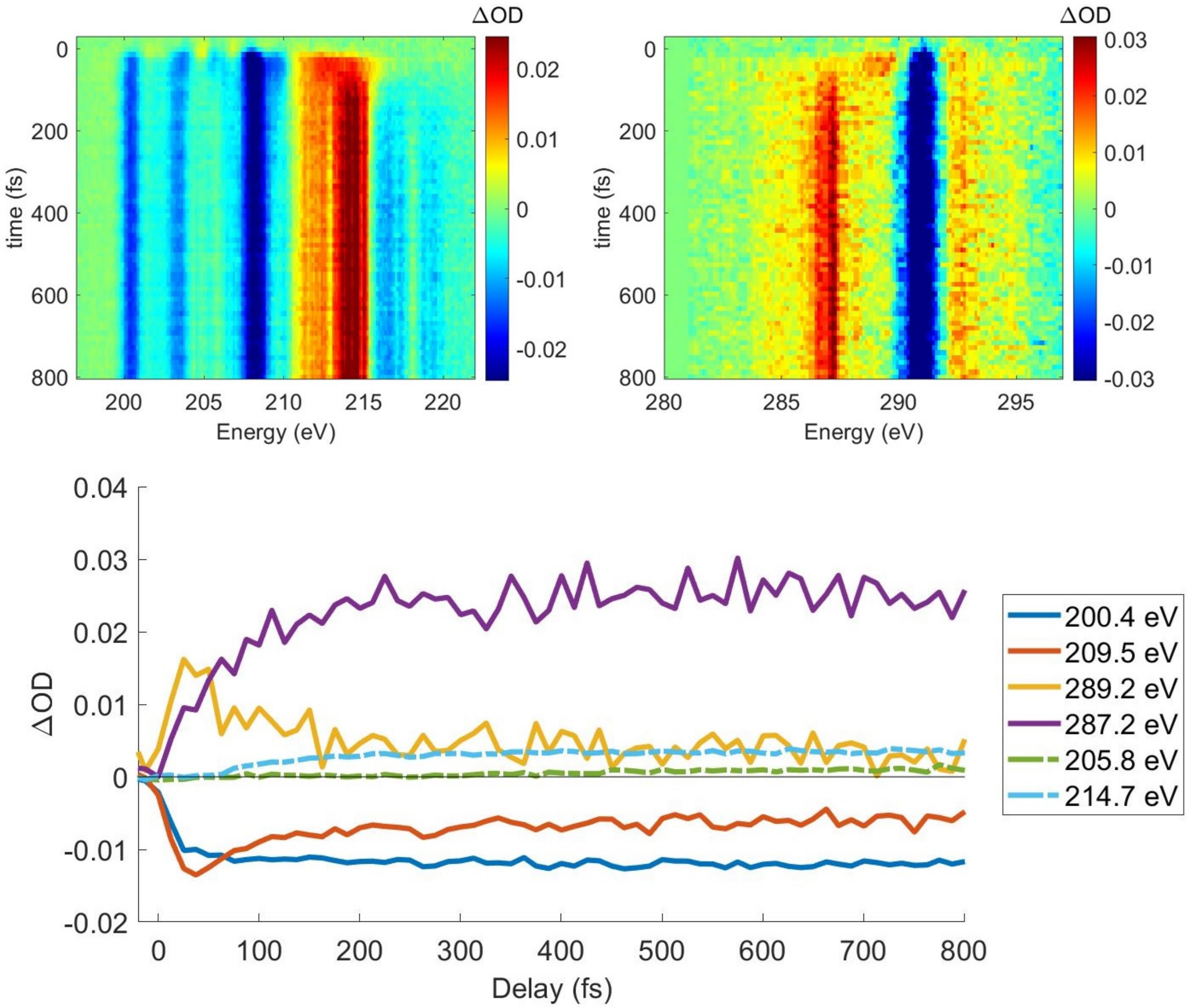

FIG. 12. Experimental data taken out to 800 fs with 12 fs steps. Maximum power is used here, $\sim 3\times10^{14}$ W/cm^2. The dashed lines of 205.8 eV and 214.7 eV are taken after applying a high pass spectral filter to the ΔOD data to isolate the parts that come from only the sharp spectral lines of the atomic Cl and Cl^+, respectively. These lineouts must be curated manually, as the energy calibration does not always correctly select the peaks, so the average energy (in the legend) may change depending on which of the multiple peaks were chosen. This is the dataset from which the $\sim$800 fs atomic Cl rise time was determined.

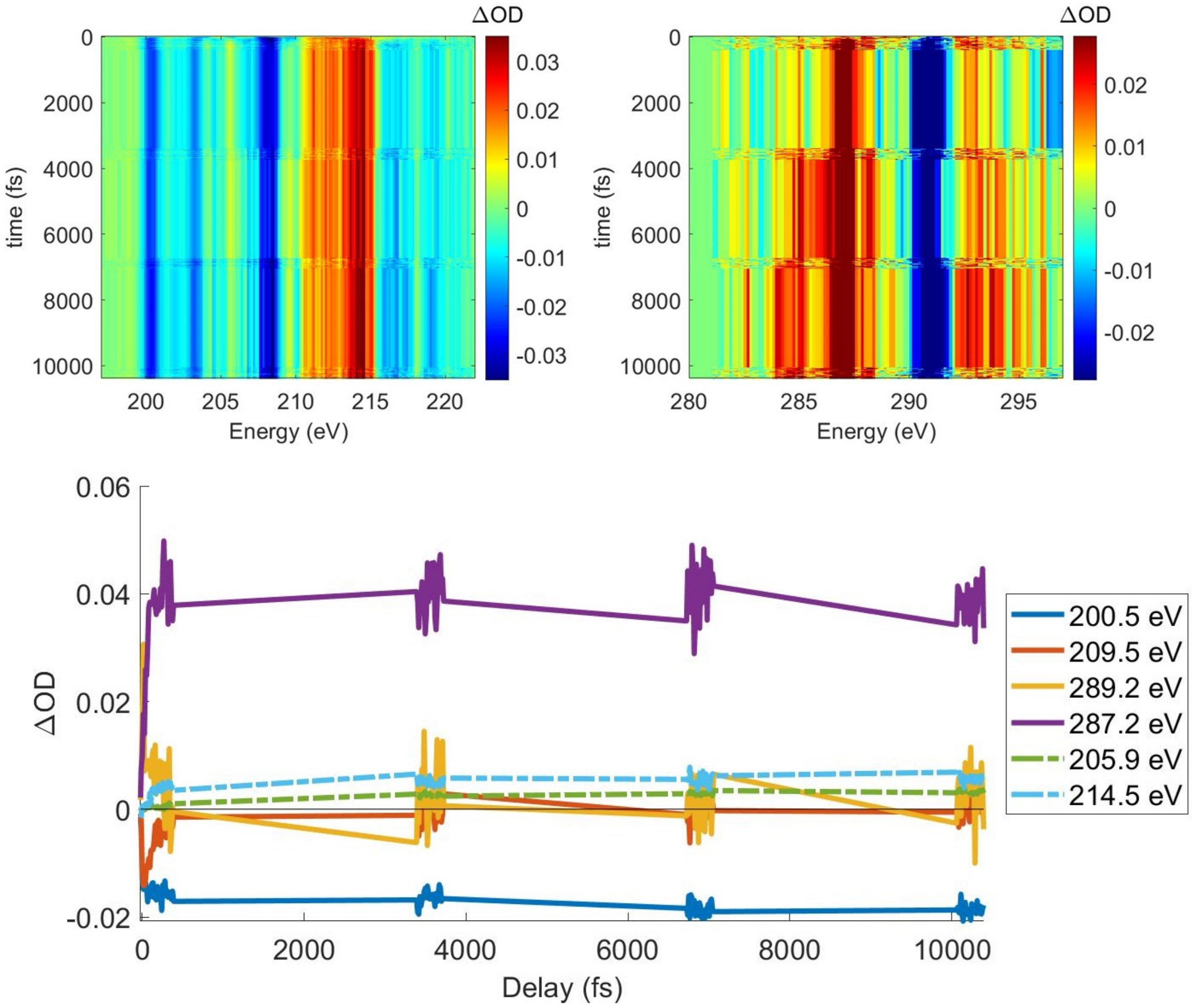

FIG. 13. Experimental data taken out to 10 ps. Data were taken in ∼300 fs blocks with 12 fs time steps with 4 blocks at 0 fs, 3.5 ps, 7 ps, and 10.5 ps in order to look at the vibrational differences between the regions. Near the maximum power is used here, 3×10^{14} W/cm^2. These are the longest delays taken in the experiment. The comparison of the atomic Cl lines are taken from the average of these data at 220 fs and 3.5 ps. The lineouts also show that the signals do not change much after the time constants given in the text, up to 10 ps.

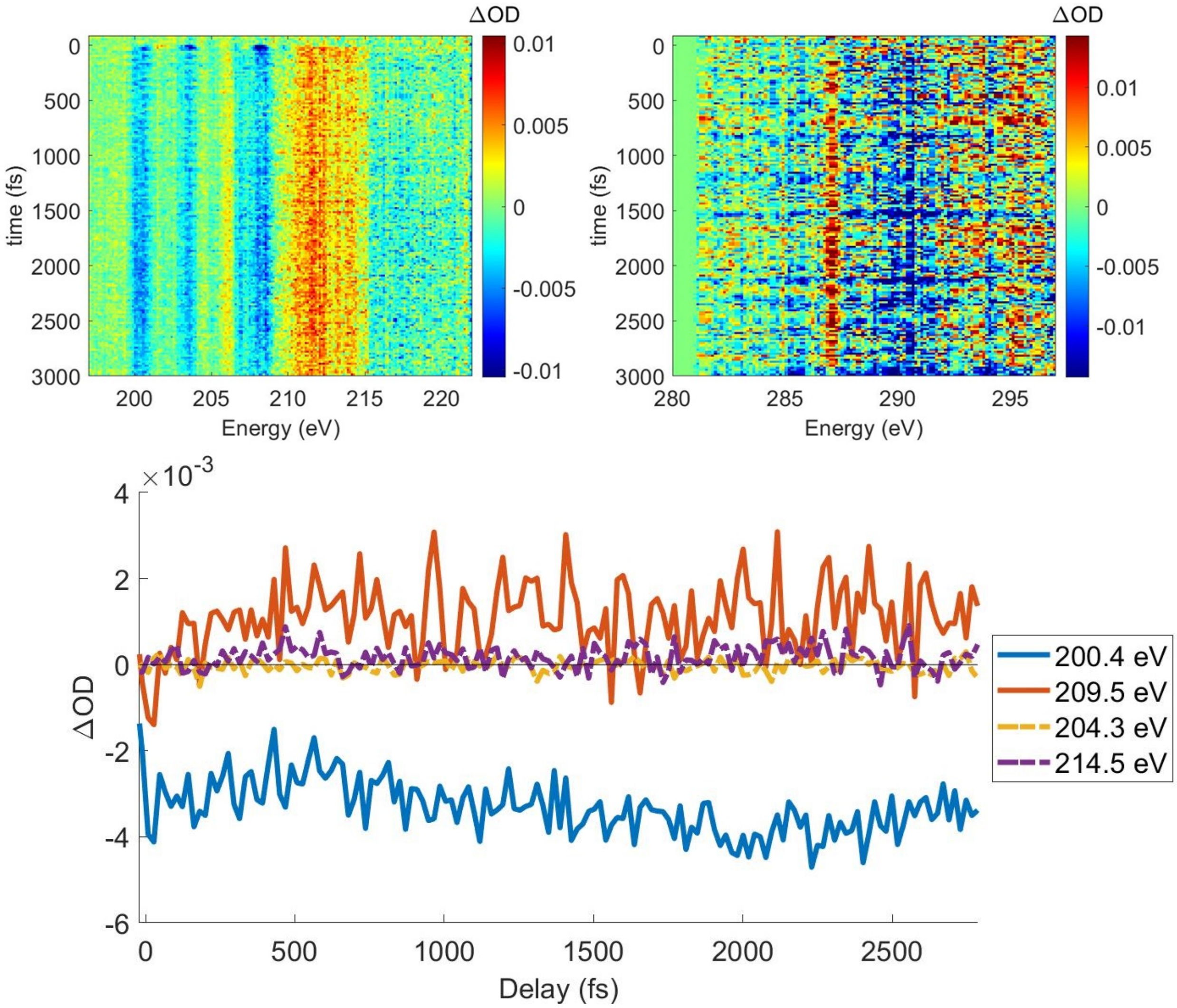

FIG. 14. Experimental data taken out to 3 ps. The focus in this scan was much looser than other datasets. Thus it represents a much lower power. No Cl formation in any significant amount is observed in this scan; although, the signals from CCl_3^+ are visible at 287.1 eV

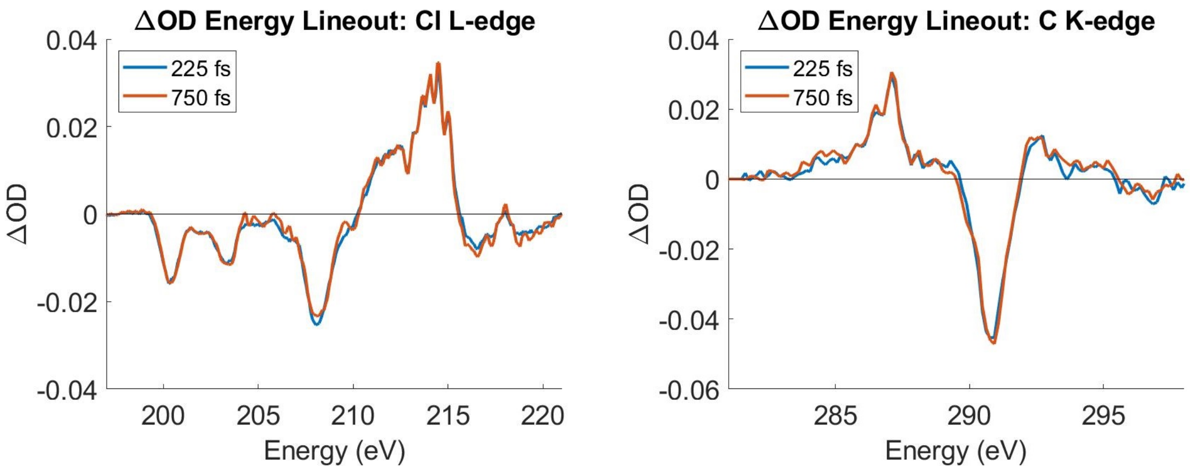

FIG. 15. Experimental data from Fig. 12 with lineouts taken at 200-250 fs and 700-800 fs at the Cl L- and C K-edges. They show that there are very little changes to the ΔOD spectra associated with the dissociation of the free Cl, other than the sharp spectral features from 204-210 eV.

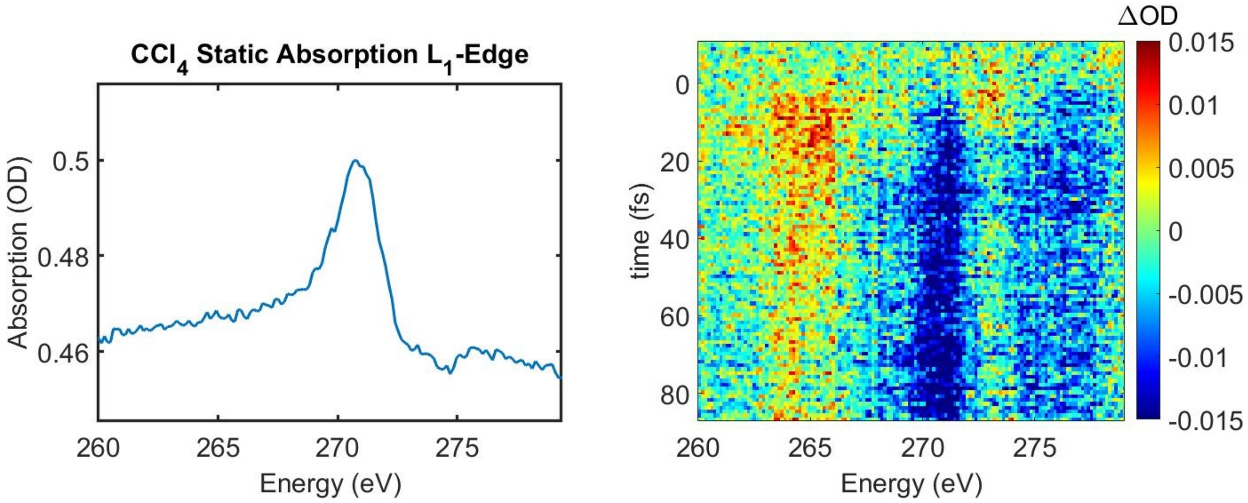

FIG. 16. Left: Static absorption of CCl_4 at the Cl L_1-edge. Only a single, broad peak can be resolved. Right: Representative transient data at the Cl L_1-edge. The data for this figure come from the same measurement as the data for Fig. 2 in the main text. This was not analyzed due to the very low signal-to-noise ratio, as compared to the other edges.

REFERENCES

[1]E. Hudson, D. A. Shirley, M. Domke, G. Remmers, A. Puschmann, T. Mandel, C. Xue, and G. Kaindl, "High-resolution measurements of near-edge resonances in the core-level photoionization spectra of sf_6," Phys. Rev. A **47**, 361–373 (1993).

[2]A. Chew, N. Douguet, C. Cariker, J. Li, E. Lindroth, X. Ren, Y. Yin, L. Argenti, W. T. Hill III, and Z. Chang, "Attosecond transient absorption spectrum of argon at the l 2, 3 edge," Physical Review A **97**, 031407 (2018).

[3]I. G. Eustatiu, T. Tyliszczak, A. P. Hitchcock, C. C. Turci, A. B. Rocha, and C. E. Bielschowsky, "Experimental and theoretical study of generalized oscillator strengths for c 1s and o 1s excitations in co_2," Phys. Rev. A **61**, 042505 (2000).

[4]R. Géneaux, H.-T. Chang, A. M. Schwartzberg, and H. J. B. Marroux, "Source noise suppression in attosecond transient absorption spectroscopy by edge-pixel referencing," Opt. Express **29**, 951–960 (2021).

[5]N. E. Henriksen and F. Y. Hansen, *Theories of Molecular Reaction Dynamics: The Microscopic Foundation of Chemical Kinetics* (Oxford University Press, USA, 2019).

[6]M. Miranda, P. Rudawski, C. Guo, F. Silva, C. L. Arnold, T. Binhammer, H. Crespo, and A. L'Huillier, "Ultrashort laser pulse characterization from dispersion scans: a comparison with spider," in *CLEO: Science and Innovations* (Optical Society of America, 2013) pp. JTh2A–31.

[7]F. Silva, M. Miranda, B. Alonso, J. Rauschenberger, V. Pervak, and H. Crespo, "Simultaneous compression, characterization and phase stabilization of gw-level 1.4 cycle vis-nir femtosecond pulses using a single dispersion-scan setup," Optics express **22**, 10181–10191 (2014).

[8]C. Ott, A. Kaldun, L. Argenti, P. Raith, K. Meyer, M. Laux, Y. Zhang, A. Blättermann, S. Hagstotz, T. Ding, *et al.*, "Reconstruction and control of a time-dependent two-electron wave packet," Nature **516**, 374 (2014).

[9]W. Cao, E. R. Warrick, D. M. Neumark, and S. R. Leone, "Attosecond transient absorption of argon atoms in the vacuum ultraviolet region: line energy shifts versus coherent population transfer," New Journal of Physics **18**, 013041 (2016).

[10]M. Y. Ivanov, M. Spanner, and O. Smirnova, "Anatomy of strong field ionization," Journal of Modern Optics **52**, 165–184 (2005), https://doi.org/10.1080/09500340420002753600.

[11]H. Ibrahim, B. Wales, S. Beaulieu, B. E. Schmidt, N. Thiré, E. P. Fowe, É. Bisson, C. T. Hebeisen, V. Wanie, M. Giguére, *et al.*, "Tabletop imaging of structural evolutions in chemical reactions demonstrated for the acetylene cation," Nature communications **5**, 1–8 (2014).

[12]X. Hao, Y. Bai, C. Li, J. Zhang, W. Li, W. Yang, M. Liu, and J. Chen, "Recollision of excited electron in below-threshold nonsequential double ionization," Communications Physics **5**, 1–7 (2022).

[13]S. Haan, L. Breen, A. Karim, and J. Eberly, "Recollision dynamics and time delay in strong-field double ionization," Optics Express **15**, 767–778 (2007).

[14]O. Smirnova, S. Patchkovskii, Y. Mairesse, N. Dudovich, and M. Y. Ivanov, "Strong-field control and spectroscopy of attosecond electron-hole dynamics in molecules," Proceedings of the National Academy of Sciences **106**, 16556–16561 (2009).

[15]J. Li, J. Lu, A. Chew, S. Han, J. Li, Y. Wu, H. Wang, S. Ghimire, and Z. Chang, "Attosecond science based on high harmonic generation from gases and solids," Nature Communications **11**, 1–13 (2020).

[16]W. Lai and C. Guo, "Polarization and molecular-orbital dependence of strong-field enhanced ionization," Physical Review A **93**, 043401 (2016).

[17]D. Mathur, K. Dota, A. Dharmadhikari, and J. Dharmadhikari, "Carrier-envelope-phase effects in ultrafast strong-field ionization dynamics of multielectron systems: Xe and cs 2," Physical Review Letters **110**, 083602 (2013).

[18]R. J. Levis and H. A. Rabitz, "Closing the loop on bond selective chemistry using tailored strong field laser pulses," The Journal of Physical Chemistry A **106**, 6427–6444 (2002), https://doi.org/10.1021/jp0134906.

[19]B. Wolter, M. G. Pullen, M. Baudisch, M. Sclafani, M. Hemmer, A. Senftleben, C. D. Schröter, J. Ullrich, R. Moshammer, and J. Biegert, "Strong-field physics with mid-ir fields," Physical Review X **5**, 021034 (2015).

[20]N. L. Wagner, A. Wüest, I. P. Christov, T. Popmintchev, X. Zhou, M. M. Murnane, and H. C. Kapteyn, "Monitoring molecular dynamics using coherent electrons from high harmonic generation," Proceedings of the National Academy of Sciences **103**, 13279–13285 (2006).

[21]Q. Song, Z. Li, H. Li, P. Lu, X. Gong, Q. Ji, K. Lin, W. Zhang, J. Ma, H. Zeng, *et al.*, "Orientation-dependent strong-field dissociative single ionization of co," Optics Express

25, 2221–2227 (2017).

[22]C. E. M. Gonçalves, R. D. Levine, and F. Remacle, "Ultrafast geometrical reorganization of a methane cation upon sudden ionization: an isotope effect on electronic non-equilibrium quantum dynamics," Phys. Chem. Chem. Phys. **23**, 12051–12059 (2021).

[23]J. Harvey, R. P. Tuckett, and A. Bodi, "Shining new light on the multifaceted dissociative photoionisation dynamics of CCl4," Physical Chemistry Chemical Physics **16**, 20492–20499 (2014).

[24]C. D. Caldwell, M. O. Krause, R. D. Cowan, A. Menzel, S. B. Whitfield, S. Hallman, S. P. Frigo, and M. C. Severson, "Inner-shell photoexcitation in an open-shell atom: The cl 2 $\vec{p}\ ns, md$ spectrum as a case study," Phys. Rev. A **59**, R926–R929 (1999).

[25]R. V. Krems, *Molecules in electromagnetic fields: from ultracold physics to controlled chemistry* (John Wiley & Sons, 2018).

[26]B. G. Lindsay, K. F. McDonald, W. S. Yu, R. F. Stebbings, and F. B. Yousif, "Electron-impact ionization of CCl_4 and CCl_2F_2," The Journal of Chemical Physics **121**, 1350–1356 (2004), https://doi.org/10.1063/1.1761055.

[27]T. Kinugawa, Y. Hikosaka, A. M. Hodgekins, and J. H. D. Eland, "New results on the dissociative photoionization of CF_4 and CCl_4," Journal of Mass Spectrometry **37**, 854–857 (2002).

[28]G. Burton, W. Chan, G. Cooper, and C. Brion, "Valence-shell and inner-shell (cl 2p, 2s, c 1s) photoabsorption and photoionization of carbon-tetrachloride - absolute oscillator-strengths (5-400 ev) and dipole-induced breakdown pathways," CHEMICAL PHYSICS **181**, 147–172 (1994).

[29]D. P. M. Geissler, *Concerted elimination of $Cl_2{}^+$ from CCl_4 and of $I_2{}^+$ from CH_2I_2 driven by intense ultrafast laser pulses*, Ph.D. thesis, The Graduate School, Stony Brook University: Stony Brook, NY. (2007).

[30]N. Mardirossian and M. Head-Gordon, "ω B97M-V: A combinatorially optimized, range-separated hybrid, meta-GGA density functional with VV10 nonlocal correlation," J. Chem. Phys. **144**, 214110 (2016).

[31]F. Jensen, "Unifying general and segmented contracted basis sets. segmented polarization consistent basis sets," J. Chem. Theory Comput. **10**, 1074–1085 (2014).

[32]K. Raghavachari, G. W. Trucks, J. A. Pople, and M. Head-Gordon, "A fifth-order perturbation comparison of electron correlation theories," Chemical Physics Letters **157**, 479–483

(1989).

[33]T. Helgaker, W. Klopper, H. Koch, and J. Noga, "Basis-set convergence of correlated calculations on water," J. Chem. Phys **106**, 9639–9646 (1997).

[34]T. H. Dunning Jr, "Gaussian basis sets for use in correlated molecular calculations. i. the atoms boron through neon and hydrogen," J. Chem. Phys. **90**, 1007–1023 (1989).

[35]R. A. Kendall, T. H. Dunning Jr, and R. J. Harrison, "Electron affinities of the first-row atoms revisited. systematic basis sets and wave functions," J. Chem. Phys. **96**, 6796–6806 (1992).

[36]D. E. Woon and T. H. Dunning Jr, "Gaussian basis sets for use in correlated molecular calculations. iii. the atoms aluminum through argon," J. Chem. Phys. **98**, 1358–1371 (1993).

[37]D. E. Woon and T. H. Dunning Jr, "Gaussian basis sets for use in correlated molecular calculations. v. core-valence basis sets for boron through neon," J. Chem. Phys. **103**, 4572–4585 (1995).

[38]K. A. Peterson and T. H. Dunning Jr, "Accurate correlation consistent basis sets for molecular core–valence correlation effects: The second row atoms al–ar, and the first row atoms b–ne revisited," J. Chem. Phys. **117**, 10548–10560 (2002).

[39]T. Shimanouchi *et al.*, *Tables of molecular vibrational frequencies* (US Government Printing Office, 1973).

[40]D. Hait and M. Head-Gordon, "Orbital optimized density functional theory for electronic excited states," J. Phys. Chem. Lett. **12**, 4517–4529 (2021).

[41]J. Sun, A. Ruzsinszky, and J. P. Perdew, "Strongly Constrained and Appropriately Normed Semilocal Density Functional," Phys. Rev. Lett. **115**, 036402 (2015).

[42]D. Hait and M. Head-Gordon, "Highly accurate prediction of core spectra of molecules at density functional theory cost: Attaining sub-electronvolt error from a restricted open-shell kohn–sham approach," J. Phys. Chem. Lett. **11**, 775–786 (2020).

[43]D. Hait, E. A. Haugen, Z. Yang, K. J. Oosterbaan, S. R. Leone, and M. Head-Gordon, "Accurate prediction of core-level spectra of radicals at density functional theory cost via square gradient minimization and recoupling of mixed configurations," J. Chem. Phys. **153**, 134108 (2020).

[44]D. Hait and M. Head-Gordon, "Excited state orbital optimization via minimizing the square of the gradient: General approach and application to singly and doubly excited

states via density functional theory," J. Chem. Theory Comput. **16**, 1699–1710 (2020).

[45]G. M. Barca, A. T. Gilbert, and P. M. W. Gill, "Simple models for difficult electronic excitations," J. Chem. Theory Comput. **14**, 1501–1509 (2018).

[46]M. A. Ambroise and F. Jensen, "Probing basis set requirements for calculating core ionization and core excitation spectroscopy by the δ self-consistent-field approach," J. Chem. Theory Comput. **15**, 325–337 (2018).

[47]M. Karplus, R. N. Porter, and R. D. Sharma, "Exchange reactions with activation energy. i. simple barrier potential for (h, h2)," J. Chem. Phys. **43**, 3259–3287 (1965).

[48]W. H. Miller, W. L. Hase, and C. L. Darling, "A simple model for correcting the zero point energy problem in classical trajectory simulations of polyatomic molecules," J. Chem. Phys. **91**, 2863–2868 (1989).

[49]D. L. Azevedo, M. H. Bettega, L. G. Ferreira, and M. A. Lima, "Scattering of low-energy electrons by ticl4, gecl4, sicl4 and ccl4: a comparison of elastic cross sections," J. Phys. B: At. Mol. Opt. Phys. **33**, 5467 (2000).

[50]Y. Pertot, C. Schmidt, M. Matthews, A. Chauvet, M. Huppert, V. Svoboda, A. von Conta, A. Tehlar, D. Baykusheva, J.-P. Wolf, and H. J. Wörner, "Time-resolved x-ray absorption spectroscopy with a water window high-harmonic source," Science **355**, 264–267 (2017), https://www.science.org/doi/pdf/10.1126/science.aah6114.

[51]M. Epshtein, V. Scutelnic, Z. Yang, T. Xue, M. L. Vidal, A. I. Krylov, S. Coriani, and S. R. Leone, "Table-top x-ray spectroscopy of benzene radical cation," The Journal of Physical Chemistry A **124**, 9524–9531 (2020).

[52]S. Larochelle, A. Talebpour, and S. Chin, "Coulomb effect in multiphoton ionization of rare-gas atoms," Journal of Physics B: Atomic, Molecular and Optical Physics **31**, 1215 (1998).

[53]R. Wang, Q. Zhang, D. Li, S. Xu, P. Cao, Y. Zhou, W. Cao, and P. Lu, "Identification of tunneling and multiphoton ionization in intermediate keldysh parameter regime," Opt. Exp. **27**, 6471–6482 (2019).
